# Single-cell analysis reveals transcriptomic and epigenomic impacts on the maternal–fetal interface following SARS-CoV-2 infection

**DOI:** 10.1038/s41556-023-01169-x

**Published:** 2023-07-03

**Authors:** Lin Gao, Vrinda Mathur, Sabrina Ka Man Tam, Xuemeng Zhou, Ming Fung Cheung, Lu Yan Chan, Guadalupe Estrada-Gutiérrez, Bo Wah Leung, Sakita Moungmaithong, Chi Chiu Wang, Liona C. Poon, Danny Leung

**Affiliations:** 1 Division of Life Science, The Hong Kong University of Science and Technology, Clear Water Bay, Hong Kong, China; 2 Center for Epigenomics Research, The Hong Kong University of Science and Technology, Clear Water Bay, Hong Kong, China; 3 Research Division, National Institute of Perinatology, Mexico City, Mexico; 4 Department of Obstetrics and Gynaecology, The Chinese University of Hong Kong, Shatin, Hong Kong, China; 5 Li Ka Shing Institute of Health Sciences; School of Biomedical Sciences and The Chinese University of Hong Kong–Sichuan University Joint Laboratory in Reproductive Medicine, The Chinese University of Hong Kong, Shatin, Hong Kong, China

**Keywords:** Epigenomics, Embryogenesis

## Abstract

During pregnancy the maternal–fetal interface plays vital roles in fetal development. Its disruption is frequently found in pregnancy complications. Recent studies show increased incidences of adverse pregnancy outcomes in patients with COVID-19; however, the mechanism remains unclear. Here we analysed the molecular impacts of SARS-CoV-2 infection on the maternal–fetal interface. Generating bulk and single-nucleus transcriptomic and epigenomic profiles from patients with COVID-19 and control samples, we discovered aberrant immune activation and angiogenesis patterns in distinct cells from patients. Surprisingly, retrotransposons were also dysregulated in specific cell types. Notably, reduced enhancer activities of LTR8B elements were functionally linked to the downregulation of pregnancy-specific glycoprotein genes in syncytiotrophoblasts. Our findings revealed that SARS-CoV-2 infection induced substantial changes to the epigenome and transcriptome at the maternal–fetal interface, which may be associated with pregnancy complications.

## Abstract (web summary)

Gao, Mathur, Tam and colleagues characterize transcriptomic and epigenomic alterations at the maternal–fetal interface in patients with COVID-19, thereby providing insights into aberrant pregnancy outcomes associated with SARS-COV-2 infection.

## Main

The coronavirus disease 2019 (COVID-19) pandemic, brought on by severe acute respiratory syndrome coronavirus 2 (SARS-CoV-2) infection, has afflicted more than 760 million people (WHO (https://covid19.who.int), May 2023). Infection during pregnancy is associated with adverse outcomes including preeclampsia, pre-term birth and stillbirth^1,2^. Although subpopulations of trophoblasts co-express the angiotensin-converting enzyme 2 (ACE2) receptor and transmembrane serine protease 2 (TMPRSS2) in early pregnancy^3,4^, placental infection and vertical viral transmission are rare^5^. Therefore, pregnancy complications are likely to result from maternal immune responses.

During pregnancy a dynamic balance is maintained between pathogen defence and preserving the fetus. This primarily occurs at the maternal–fetal interface (MFI), where fetal cells extensively invade and interact with maternal decidua^6^. About 40% of the maternal decidual cells during early pregnancy are immune cell types^7^. Altered composition and functionality of these cells underlie various pathologies^8,9^.

In the placenta retrotransposons provide an abundant source of functional sequences for host genomes^10^. Retrovirus-derived genes, including the *SYNCYTIN*s, are necessary for normal placenta development and function^11–13^. Moreover, retrotransposons pervasively shape placental *cis*-regulatory element (CRE) landscapes across species^14–16^. Strikingly, viral infections, including SARS-CoV-2, are associated with aberrant derepression of retrotransposons^17^, which may cause widespread transcriptional dysregulation.

The molecular mechanism of COVID-19-associated pregnancy complications is unclear. Given the complex makeup of the MFI, cell type-specific analysis is necessary. Although there have been single-nucleus transcriptomic studies on placental samples from patients infected with COVID-19 (refs. ^18–20^), the associated epigenomic alterations remain unknown. Here we investigated the cell type-specific molecular dysregulation at the MFI in patients infected with SARS-CoV-2 by mapping the transcriptomes and epigenomes at both bulk and single-nucleus resolutions. We detected global transcriptomic and epigenomic changes in patients, which included misregulation of immune-response and angiogenesis genes. Interestingly, we discovered dysregulated retrotransposons, specifically LTR8B-derived enhancers, which were linked to the reduced expression of pregnancy-specific glycoprotein (*PSG*) genes. Collectively, we generated extensive profiles from the MFI of patient and control samples, and highlighted the involvement of epigenetic regulation of CREs and retrotransposons in COVID-19-related pregnancy complications.

## Results

### Multi-omic profiling of the MFI in patients with COVID-19

**Fig. 1:**
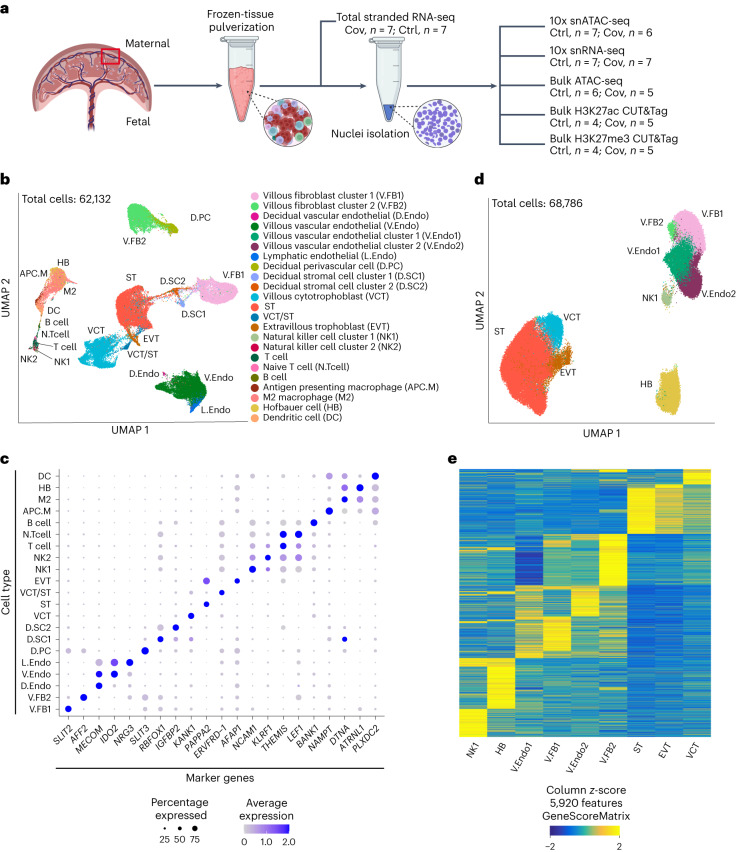
Multi-omic analysis of the MFI in patients with COVID-19. **a**, Schematic of the experiment design. Frozen tissue samples were pulverized and subjected to RNA extraction for stranded total RNA-seq. The remaining tissue powder underwent nuclei preparation for the following assays: bulk ATAC-seq, snRNA-seq, snATAC-seq and CUT&Tag for H3K27ac and H3K27me3 histone modifications. We then carried out integrative analysis on the multi-omic datasets derived from patient and control MFI samples. **b**, UMAP of the snRNA-seq showing 21 identified cell types. **c**, Bubble plot of marker genes for each of the 21 cell types identified in the snRNA-seq. The size of the bubbles represents the percentage of cells within a cluster that expresses the marker gene and the colour of the bubble represents the average expression level of the marker gene calculated by Seurat. **d**, UMAP of the snATAC-seq showing nine identified cell types. **b**,**d**, Each dot represents a nucleus and the colours correspond to its annotated cell type. **e**, Heatmap of the marker gene scores calculated by ArchR for all nine cell types in the snATAC-seq datasets. Each column represents the gene score of a marker gene and each row represents a cell type. Ctrl, control and Cov, COVID-19; the cell-type abbreviations in **c**–**e** are defined in **b**.

**Extended Data Fig. 1:**
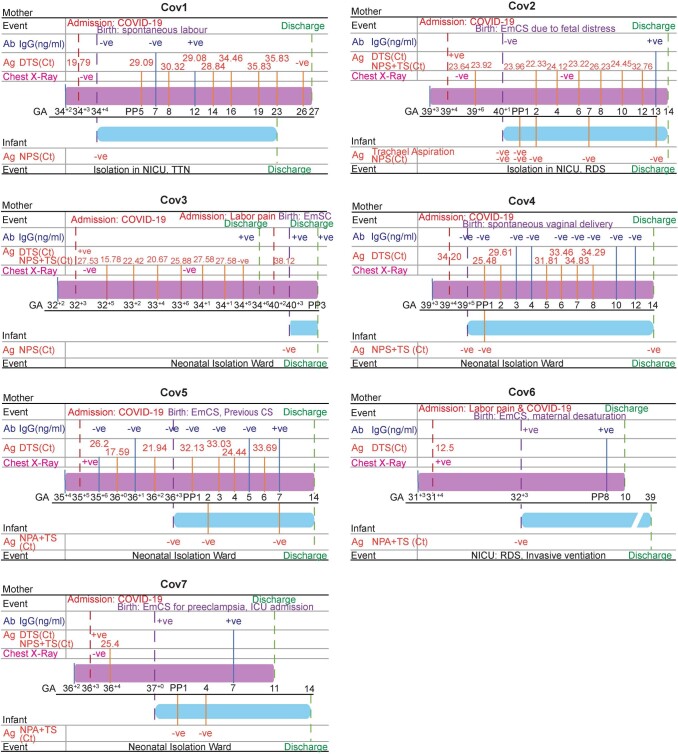
Case timelines for the patient samples. Timeline of SARS-CoV-2 infection for each COVID-19 patient participating in this study. The timelines show the relationship between infection period, hospital admission due to SAR-CoV-2 infection, hospital admission for delivery and postpartum period. Discharge, discharge from hospital; COVID-19, Coronavirus disease 2019; Ab, antibody; IgG, immunoglobulin G; Ag, Antigen; DTS, deep throat saliva; NPS, nasopharyngeal swab; NPA, nasopharyngeal aspiration; TS, throat swab; CS, Caesarean section; EmCS, emergency Caesarean section; RDS, respiratory distress syndrome; *C*_t_, cycle threshold; NSD, normal spontaneous delivery; TTN, transient tachypnea of the newborn; ICU, intensive care unit; NICU, neonatal intensive care unit; +ve, positive finding; −ve, negative finding; PP, postpartum; and GA, gestational age.

**Extended Data Fig. 2:**
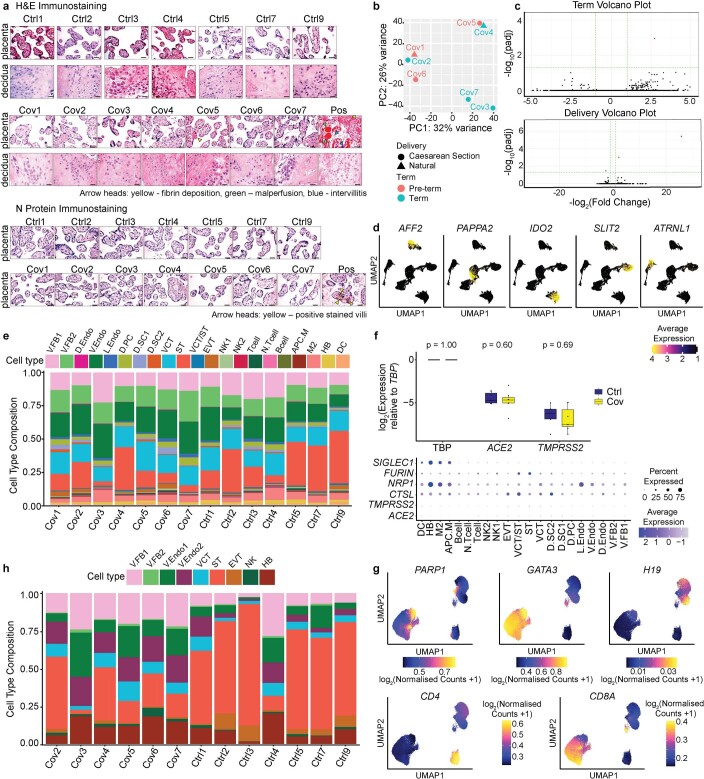
Quality control and cell-type analysis for samples and multi-omic datasets. **a**, Haematoxylin and eosin (top) histological staining and immunostaining of SARS-CoV-2 nucleocapsid protein (bottom) in patients and control tissue. Scale bars, 50 µm. No replicates were performed for the haematoxylin and eosin or N protein immunostaining. **b**, Principal component analysis (PCA) on the patient samples (*n* = 7) used in this study based on gene expression from the bulk RNA-seq. The colour of the sample indicates if the pregnancy was carried out to pre-term or term and the shape represents the mode of delivery—either CS or natural delivery. **c**, DEGs within patient samples when compared according to term of pregnancy (pre-term/term; top) and mode of delivery (CS/natural; bottom). The significance is presented as adjusted two-tailed *P* value from multiple testing using the Benjamini and Hochberg method (*P*adj) from DESeq2. Threshold, *P*adj < 0.05 and log_2_(fold change) > 1. **d**, Expression of top marker genes from the snRNA-seq calculated by Seurat. **e**, Cell-type distribution in all samples from the snRNA-seq after filtering. **f**, Expression of known SARS-CoV-2 receptors in each cell type from the snRNA-seq calculated by Seurat (bottom). The size of the bubble indicates the percentage of cells in each cell type that expresses the gene. RT–qPCR results of *ACE2* and *TMPRSS2* in our patient and control samples (top). Each dot represents a sample (*n* = 7 for both patient and control); *P* values were calculated using a one-tailed Wilcoxon test. The centre and bounds of boxes indicate the median and quartile of all data points, respectively. The minima and maxima of whiskers indicate quartile 1 − 1.5× the interquartile range and quartile 3 + 1.5× the interquartile range, respectively. **g**, Gene score of top marker genes from the snATAC-seq calculated by ArchR. **h**, Cell-type distribution in all samples from the snATAC-seq after filtering. Source data

To elucidate molecular changes at the MFI following SARS-CoV-2 infection, we assessed the transcriptome and chromatin states of patient and control samples at both bulk and single-nucleus levels (Fig. 1a). We enrolled seven patients who were infected with SARS-CoV-2 during pregnancy and seven healthy pregnant donors (Extended Data Fig. 1 and Supplementary Tables 1 and 2). The patients tested positive for SARS-CoV-2 by quantitative PCR with reverse transcription (RT–qPCR; *C*_t_ ≤ 35) during late pregnancy (31.6–39.6 weeks). Among the patients, six displayed mild symptoms and one had severe symptoms, requiring admission to the intensive care unit and invasive ventilation. All MFI samples tested negative for SARS-CoV-2 by RT–qPCR (Supplementary Table 3) and as well as N protein staining (Extended Data Fig. 2a). We performed sensitivity analyses to confirm that factors including modes of delivery and gestational ages at delivery did not significantly impact the transcriptomic profiles (Extended Data Fig. 2b,c).

We conducted single-nucleus RNA-sequencing (snRNA-seq) on all patient and control samples (Supplementary Table 4). After filtering we analysed 62,132 nuclei (patients, *n* = 27,480 and controls, *n* = 34,652). Sequencing reads from each nucleus were mapped to both the human GRCh38/hg38 reference and the SARS-CoV-2 genomes^21^. To delineate cell-type identities, we performed unsupervised clustering with uniform manifold approximation and projection (UMAP). We identified 21 distinct cell types in the patient and control samples, which aligned with known cell categories at the MFI: trophoblasts, T cells, B cells, NK cells, macrophages, dendritic cells, endothelial cells, perivascular cells, fibroblasts and stroma cells (Fig. 1b,c, Extended Data Fig. 2d,e and Supplementary Fig. 1a). Concordant with the RT–qPCR and staining results, we found no detectable enrichment of viral transcripts in any samples (Supplementary Fig. 1b). Consistent with recent findings^18^, *ACE2* and *TMPRSS2* were expressed at low-to-undetectable levels in all term samples (Extended Data Fig. 2f).

To investigate gene regulation, we mapped the chromatin accessibility at the MFI by single-nucleus assay for transposase-accessible chromatin with sequencing (snATAC-seq; Supplementary Table 4). We profiled 68,786 individual nuclei from six patients and seven control samples (patients, *n* = 22,926 and controls, *n* = 45,860; Fig. 1a). Based on these maps, we defined nine cell types, including trophoblasts, immune cells, endothelial cells and fibroblasts (Fig. 1d). Each cell type showed distinct open chromatin patterns at the transcriptional start sites of marker genes (Fig. 1e and Extended Data Fig. 2g). Similar to snRNA-seq, syncytiotrophoblasts (STs) were the most abundantly profiled (Extended Data Fig. 2h).

To increase the depth of our epigenomic analysis, we also conducted RNA-seq, ATAC-seq and CUT&Tag targeting acetylation and tri-methylation of histone H3 lysine 27 (H3K27ac and H3K27me3) modifications on bulk patient and control samples (Fig. 1a and Supplementary Table 4). H3K27ac is a key histone modification found at active CREs and H3K27me3 is an important repressive mark regulating development genes^22^. We investigated whether epigenomic pathways are impacted by COVID-19. We observed a high correlation between the pseudo-bulk single-nucleus and bulk assays, confirming reproducibility between different data modalities (Supplementary Fig. 1c,d and Supplementary Data). Together, we generated extensive single-nucleus and bulk multi-omic profiles of the MFI from control and SARS-CoV-2-infected study participants, which we utilized for subsequent integrative analyses.

### COVID-19 impacts the transcriptome and epigenome of the MFI

**Fig. 2:**
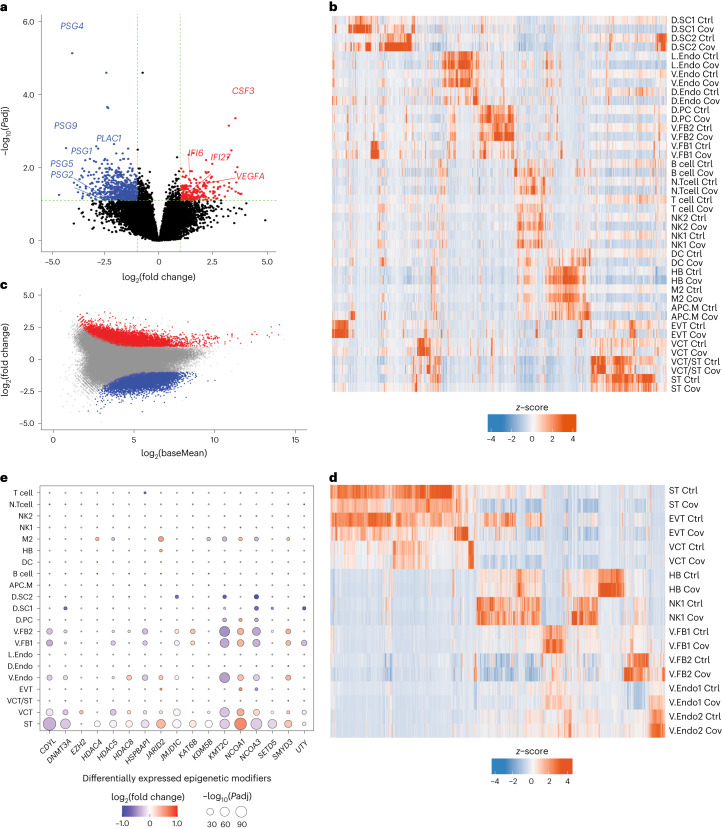
SARS-CoV-2 infection is associated with transcriptomic and epigenomic dysregulation. **a**, Dysregulated genes from the bulk RNA-seq. Each dot represents a gene. The negative log_10_-transformed two-tailed Wald test *P* values adjusted for multiple testing using the Benjamini and Hochberg method (*P*adj) and log_2_-transformed fold change (patient/control) were calculated by DESeq2. Upregulated (*P*adj < 0.05 and log_2_(fold change) > 1; *n* = 211) and downregulated (*P*adj < 0.05 and log_2_(fold change) < −1; *n* = 605) genes are labelled in red and blue, respectively. Dashed lines represent the indicated thresholds. **b**, Heatmap showing dysregulated genes from the snRNA-seq. For each cell type, upregulated and downregulated genes (one-tailed MAST model *P*adj < 0.05 and log_2_(fold change) > 0.25) were called by comparing the patient and control samples using Seurat. **c**, MA plot showing differentially accessible regions from the bulk ATAC-seq. Each dot represents a peak. The negative log_2_-transformed fold change (patient/control) and log_2_(average signal, baseMean) were calculated by DESeq2. Increased (two-tailed Wald test *P*adj < 0.01 and log_2_(fold change) > 1; *n* = 8,223) and decreased (two-tailed Wald test *P*adj < 0.001 and log_2_(fold change) < −1; *n* = 7,142) peaks are labelled in red and blue, respectively. **d**, Differentially accessible regions from the snATAC-seq. For each cell type, differential peaks (one-tailed Poisson test *P*adj < 0.01, fold change > 1.5 and reads per kilobase per million mapped reads (RPKM) > 1 in the tested group) were called by comparing the patient and control samples. **b**,**d**, Each row represents a cell type from a patient or control and each column represents a gene (**b**) or differentially accessible region (**d**). The colour scale indicates the calculated average gene expression (**b**) or RPKM (**d**) column *z*-score. **e**, Epigenetic modifiers that were differentially expressed between patient and control samples for each cell type from the snRNA-seq. The *P*adj was calculated using the one-tailed MAST model from Seurat. Ctrl, control and Cov, COVID-19; cell-type abbreviations as per Fig. 1b.

**Extended Data Fig. 3:**
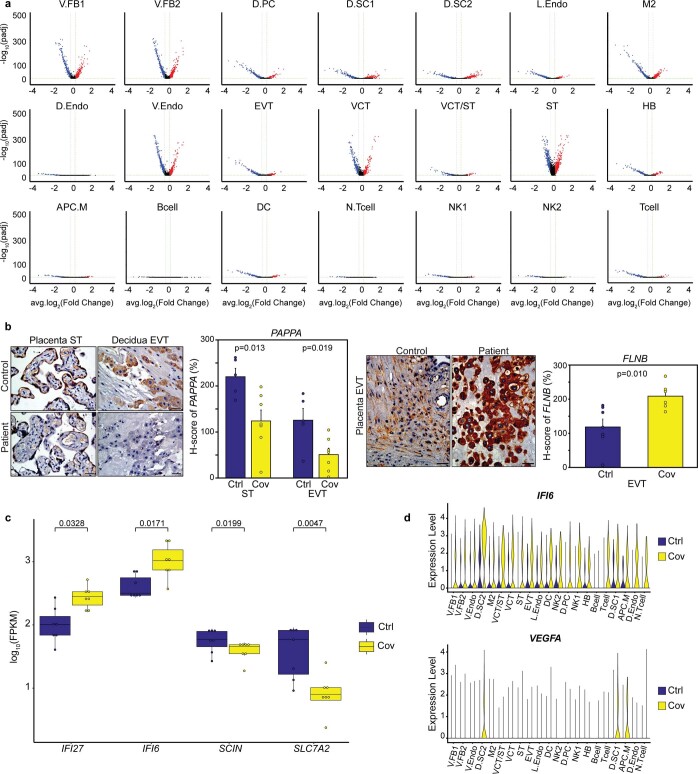
Characterization of transcriptional changes in patient samples. **a**, DEGs between patient and control samples in all 21 cell types from the snRNA-seq. Upregulated genes (one-tailed MAST model *P*adj < 0.05 and log_2_(fold change) > 0.25) and downregulated genes (one-tailed MAST model *P*adj < 0.05 and log_2_(fold change) < −0.25) are displayed in red and blue, respectively. **b**, Immunohistochemistry staining (left) and the corresponding H-score (right) of The DEGs *PAPPA* and *FLNB* in placenta and decidua tissue. Scale bars, 50 µm. Each dot in the bar chart represents a sample (*PAPPA*, *n* = 7 for patient and *n* = 5 for control; *FLNB*, *n* = 7 for patient and *n* = 7 for control). Data are presented as the mean with error bars showing the s.e.m.; *P* values were calculated using a two-tailed Student’s *t*-test. **c**, Boxplots showing the bulk RNA-seq FPKM signal of DEGs defined by the snRNA-seq for all samples. Each dot represents a sample (*n* = 7 for both patient and control); a two-tailed Wald test *P*adj was calculated from DESeq2. The centre and bounds of boxes indicate the median and quartile of all data points, respectively. The minima and maxima of whiskers indicate quartile 1 − 1.5× the interquartile range and quartile 3 + 1.5× the interquartile range, respectively. **d**, Expression of *IFI6* (top) and *VEGFA* (bottom) for each cell type from the snRNA-seq calculated by Seurat. Source data

Next, we investigated the cell type-specific molecular changes at the MFI following SARS-CoV-2 infection (Supplementary Tables 5 and 6). We identified hundreds of differentially expressed genes (DEGs) from the bulk RNA-seq of patients (Fig. 2a; upregulated, *n* = 211 and downregulated, *n* = 605), suggesting extensive transcriptomic dysregulation. We discovered downregulation of placenta developmental and pregnancy-related genes, including *PLAC1* and several *PSG* genes, whereas immune-related and angiogenesis genes such as *IFI6*, *IFI27*, *CSF3* and *VEGFA* were upregulated (Fig. 2a). From snRNA-seq, we further found cell type-specific DEGs (Fig. 2b and Extended Data Fig. 3a). Selected DEGs were validated by immunohistochemical staining (Extended Data Fig. 3b). We noted a high consistency of DEG expression across individual patients (Extended Data Fig. 3c). As expected, some DEGs identified in the bulk data were ubiquitously dysregulated across different cell types—for example, *IFI6*—whereas others were contributed by specific cell types—for example, the upregulation of *VEGFA* was mainly observed in stroma cells and fibroblasts (Extended Data Fig. 3d and Supplementary Fig. 2a).

**Extended Data Fig. 4:**
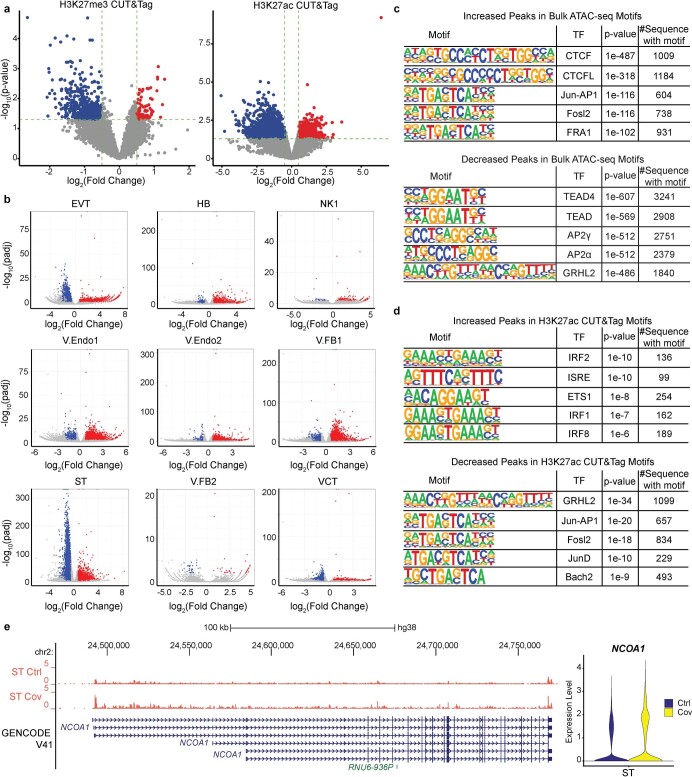
Characterization of epigenomic changes in patient samples. **a**, Differential peaks from the H3K27me3 (left) and H3K27ac (right) CUT&Tag data. The fold change and two-tailed Wald test *P* values were calculated by DESeq2. The H3K27me3 (left) volcano plot shows increased peaks (*P* < 0.05 and log_2_(fold change) > 0.5, *n* = 77) and decreased peaks (*P* < 0.05 and log_2_(fold change) < −0.5, *n* = 502) in red and blue, respectively. The H3K27ac (right) volcano plot shows increased peaks (*P* < 0.05 and log_2_(fold change) > 0.5, *n* = 816) and decreased peaks (*P* < 0.05 and log_2_(fold change) < −0.5, *n* = 2,844) in red and blue, respectively. **b**, Differential chromatin accessibility between patient and control samples in nine cell types from the snATAC-seq. Increased peaks (one-tailed Poisson test *P*adj < 0.01, log_2_(fold change) > 0.585 and RPKM > 1 in Cov) and decreased peaks (one-tailed Poisson test *P*adj < 0.01, log_2_(fold change) < −0.585 and RPKM > 1 in Ctrl) are shown in red and blue, respectively. **c**,**d**, HOMER motif analysis of both increased (top) and decreased (bottom) peaks from the bulk ATAC-seq (**c**) and the H3K27ac CUT&Tag (**d**). *P* values were calculated using a one-tailed hypergeometric test. **e**, Genome browser screenshot (left) and violin plot (right) showing the upregulation of the *NCOA1* gene in syncytiotrophoblasts (ST). The pseudo-bulk tracks of ST cells from the snRNA-seq are displayed as RPM and the *y* axes range from 0 to 5 (left). The *y* axis of the violin plot shows the expression of *NCOA1* in ST cells from the snRNA-seq calculated by Seurat (right). Source data

Epigenomic analysis revealed thousands of regions with altered chromatin accessibility or aberrant H3K27ac/H3K27me3 enrichment (Fig. 2c, Extended Data Fig. 4a and Supplementary Fig. 2b), indicating that chromatin states were impacted by SARS-CoV-2 infection. Cell type-specific differentially accessible regions were defined by snATAC-seq (Fig. 2d, Extended Data Fig. 4b and Supplementary Fig. 2c). Intriguingly, regions with increased chromatin accessibility were enriched with CTCF motifs, suggesting involvement with *cis*-regulatory higher-order chromatin interactions (Extended Data Fig. 4c). Concordant with the downregulation of pregnancy-related genes, loci with reduced chromatin accessibility or H3K27ac enrichment harboured placental transcription factor motifs, including TEAD4 and GRHL2 (Extended Data Fig. 4d). In line with the upregulation of interferon genes, regions with increased H3K27ac were enriched with motifs for interferon regulatory factors (Extended Data Fig. 4d).

To investigate the non-cell-autonomous epigenomic disruptions by COVID-19 (ref. ^23^), we analysed the expression of epigenetic modifying enzymes and identified 17 factors that were differentially expressed in at least one cell type (Fig. 2e). Concordant with H3K27ac and H3K27me3 enrichment changes in patients, we observed significant dysregulation of histone deacetylase (*HDAC*s; *HDAC4*, *HDAC5* and *HDAC8*) and H3K27 histone methyltransferase (enhancer of zeste homolog 2, *EZH2*) genes in different cell types (Fig. 2e). Notably, STs exhibited altered expression of additional chromatin modifiers, including the upregulation of nuclear receptor coactivator 1 (*NCOA1*; Fig. 2e and Extended Data Fig. 4e), which can be transcriptionally induced by interferon treatment^24^. Our results suggest that SARS-CoV-2 infection potentially disrupts host epigenetic pathways via non-cell-autonomous manners like activation of immune responses. Such transcriptomic and epigenomic effects at the MFI can influence angiogenesis and other important processes following SARS-CoV-2 infection during pregnancy.

### Aberrant immune activation at the MFI following infection

**Fig. 3:**
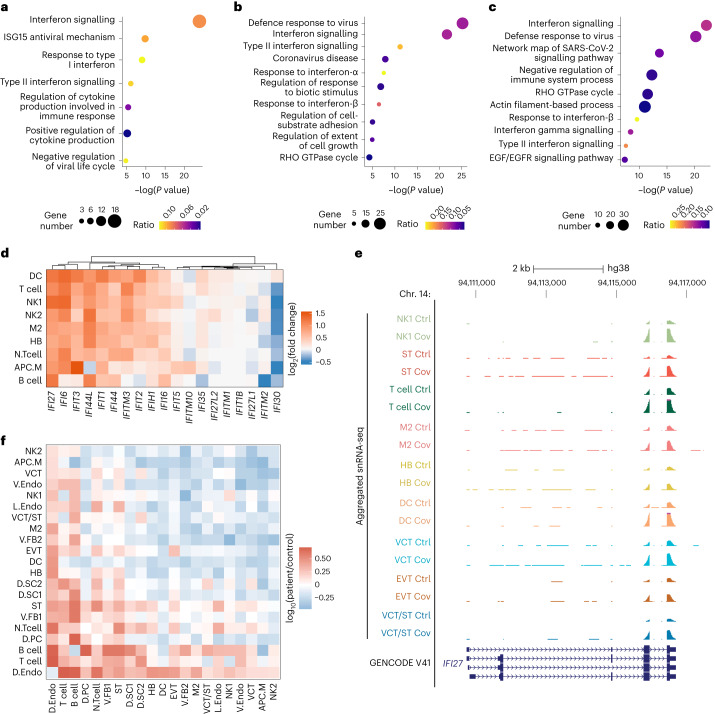
Immune activation at the MFI during SARS-CoV-2 infection. **a**–**c**, GO analysis of upregulated genes from snRNA-seq in dendritic (**a**), Hofbauer (**b**) and M2 macrophage (**c**) cells. The size and colour of the bubble represent the number of upregulated genes and the ratio to total genes under each GO term, respectively. *P* values were calculated using a one-tailed hypergeometric test. **d**, Hierarchically clustered heatmap showing the log_2_(fold change) of selected interferon-induced gene expression in immune cell types from the snRNA-seq. **e**, Genome browser screenshot of the genomic region containing *IFI27*. The pseudo-bulk tracks for patient and control samples of immune and trophoblast cell types from the snRNA-seq are displayed as reads per million (RPM). The *y* axes for all tracks range from 0 to 75. **f**, Change in CellPhoneDB receptor–ligand interactions. Each row and column correspond to a cell type from the snRNA-seq and the colour indicates the log_2_(fold change) of the number of interactions between the patient and control samples. Ctrl, control and Cov, COVID-19; cell-type abbreviations as per Fig. 1b.

**Extended Data Fig. 5:**
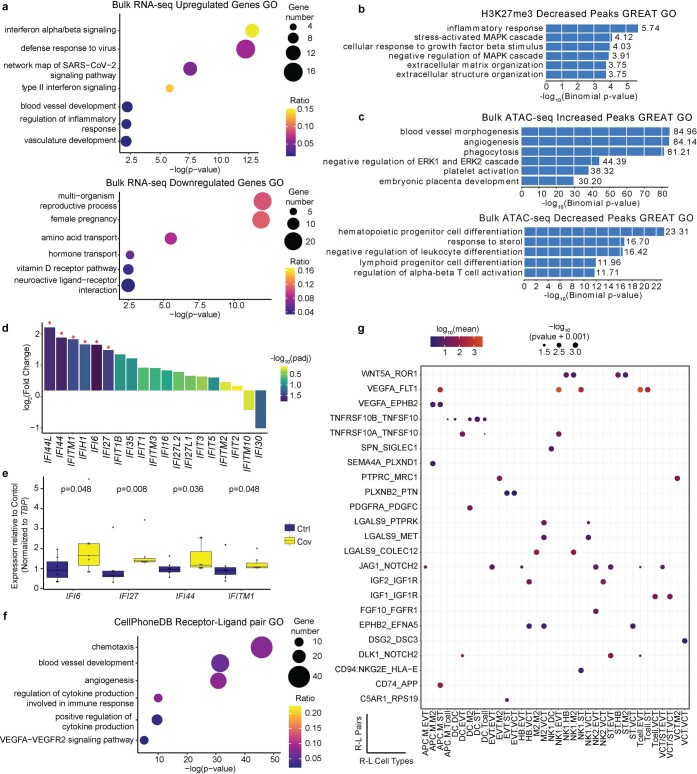
Gene ontology analysis and CellPhoneDB results for bulk and single-nucleus assays showing immune and angiogenesis dysregulation. **a**, GO analysis for upregulated (top) and downregulated genes (bottom) from the bulk RNA-seq. The colour scale represents the ratio of dysregulated genes to total genes under each GO term; *P* values were calculated using a one-tailed hypergeometric test. **b**,**c**, GREAT analysis of decreased peaks from the H3K27me3 CUT&Tag (**b**) and increased (**c**,top) and decreased (**c**,bottom) peaks from the bulk ATAC-seq. *P* values were calculated using a one-tailed binomial test. **d**, Expression of selected interferon genes from the bulk RNA-seq. The fold change (patient/control) and *P*adj using the two-tailed Wald test were calculated by DESeq2. Significantly dysregulated genes (|log_2_(fold change)| > 1 and *P*adj < 0.05) are marked by red stars. **e**, RT–qPCR results of selected interferon genes in our patient and control samples. Each dot represents a sample (*n* = 7 for both patient and control); *P* values were calculated using a one-tailed Wilcoxon test. The centre and bounds of boxes indicate the median and quartile of all data points, respectively. The minima and maxima of whiskers indicate quartile 1 − 1.5× the interquartile range and quartile 3 + 1.5× the interquartile range, respectively. **f**, GO analysis of significant patient-specific receptor–ligand interactions, defined by CellPhoneDB, in which the ligand is differentially expressed (|log_2_(fold change)| > 0.25 and one-tailed MAST model *P*adj < 0.05) in given cell types from Seurat. The colour scale represents the ratio of dysregulated genes to total genes under each GO term; *P* values were calculated using a one-tailed hypergeometric test. **g**, Significant patient-specific receptor–ligand pairs under the GO term regulation of cytokine production defined by CellPhoneDB (see Methods). Only interactions between immune and trophoblast cell types are shown. The mean expression and *P* values (one-tailed Wilcoxon test) were calculated by CellPhoneDB. Source data

Cytokine storms and adverse immune-related outcomes were previously reported in patients with COVID-19 (ref. ^25^). We investigated whether an analogous phenomenon was present at the MFI of patients. We detected upregulation of interferon-related genes in the patient samples (Fig. 2a). Correspondingly, upregulated genes were enriched with gene ontology (GO) terms relating to interferon signalling, inflammatory response and defence against viruses (Fig. 3a–c and Extended Data Fig. 5a). De-repressed loci that lost H3K27me3 in patients were associated with GO terms for inflammatory responses and interferon pathways (Extended Data Fig. 5b). Similarly, regions with increased H3K27ac were enriched with interferon regulatory factor binding motifs (Extended Data Fig. 4d). Conversely, loci with reduced chromatin accessibility were associated with GO terms related to negative regulation of immune cells (Extended Data Fig. 5c), in line with aberrant immune activation in patients.

From the snRNA-seq analysis, most interferon-inducible (*IFI*) and interferon-induced transmembrane (*IFITM*) genes were upregulated in at least one immune cell type (Fig. 3d), which was confirmed in the bulk RNA-seq and by RT–qPCR (Extended Data Fig. 5d,e). For instance, *IFI27*, which encodes for an interferon-α-inducible protein, was upregulated in most immune cell types and trophoblasts in patient samples (Figs. 2a and 3d,e). Interestingly, overexpression of *IFI27* in peripheral blood cells was reported as a potential biomarker for SARS-CoV-2 infection^26^. Our results suggest that *IFI27* upregulation could be a non-cell type-restrictive response following SARS-CoV-2 infection and is associated with epigenomic reprogramming in patients.

To further characterize the dysregulation of immune-related pathways, we employed CellPhoneDB to find alterations in receptor–ligand interactions within our snRNA-seq datasets^27^. CellPhoneDB predicts cell population interactions by measuring the expression of ligands and receptors of all known pairs across distinct cell types^27^. We detected altered interactions relative to controls in multiple cell types of patients, including interactions between different immune cell types as well as interactions between immune and other cell types, which is indicative of immune dysregulation (Fig. 3f). We then filtered for patient-specific receptor–ligand interactions in which the ligand expression is upregulated in particular cell types. Interestingly, we found those COVID-19-induced interactions to be associated with GO terms including cytokine production (Extended Data Fig. 5f,g). Together, we report a significant increase in immune response, characterized by the upregulation of interferon-induced and cytokine-signalling genes in a cell type-specific manner, at the MFI following SARS-CoV-2 infection.

### Angiogenesis dysregulation due to COVID-19 infection during pregnancy

**Fig. 4:**
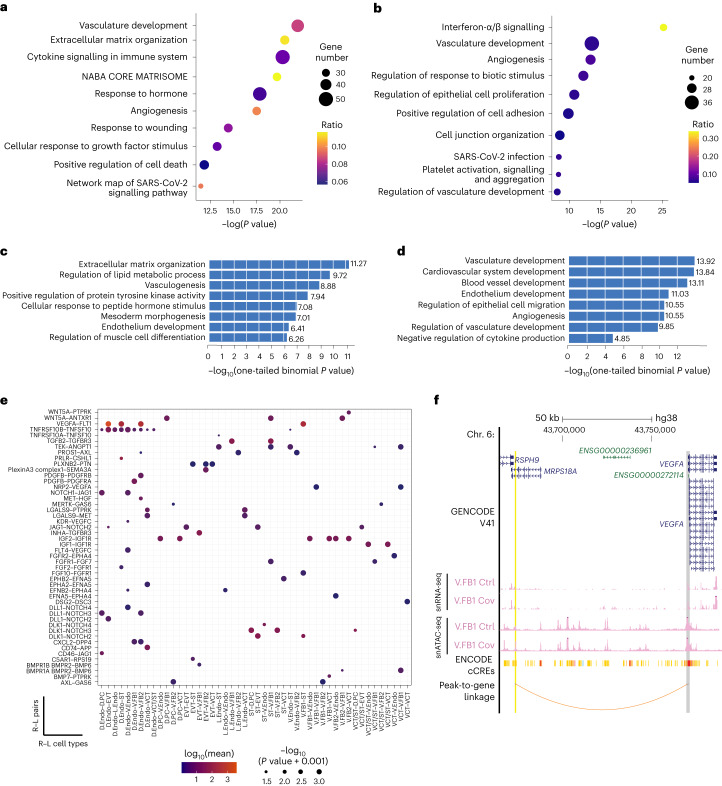
Dysregulation of angiogenesis pathways due to SARS-CoV-2 infection. **a**,**b**, GO analysis of upregulated genes from snRNA-seq in villous fibroblast cluster 1 (V.FB1; **a**) and villous vascular endothelial (V.Endo1; **b**) cells. The size and colour of the bubble represent the number of upregulated genes and the ratio to total genes under each GO term, respectively. *P* values were calculated using a one-tailed hypergeometric test. **c**,**d**, GREAT analysis of increased peaks in the V.FB1 (**c**) and V.Endo1 (**d**) cells from the snATAC-seq. The top relevant GO terms are shown. **e**, Significant patient-specific receptor–ligand (R–L) pairs under the GO term of angiogenesis. The colour of the bubbles indicates the log_10_(mean expression) and the size of the bubbles represents the significance in negative log_10_(one-tailed Wilcoxon test *P* value + 0.001) from CellPhoneDB. **f**, Genome browser screenshot of the genomic region upstream of *VEGFA*. The pseudo-bulk tracks for patient and control samples of V.FB1 from the snATAC-seq and the snRNA-seq are displayed as RPM. The grey shading highlights the promoter region of *VEGFA* and the yellow shading highlights a potential *cis*-regulatory enhancer called by the peak-to-gene linkage analysis (orange arc). This potential enhancer region is defined with significantly increased ATAC-seq signal in V.FB1 cells (fold change (COVID-19/control)) = 1.8 and one-tailed Poisson test *P*adj = 1.26 × 10^−5^). The *y* axes for all cell type tracks range from zero to two. Ctrl, control and Cov, COVID-19; cell-type abbreviations as per Fig. 1b.

**Extended Data Fig. 6:**
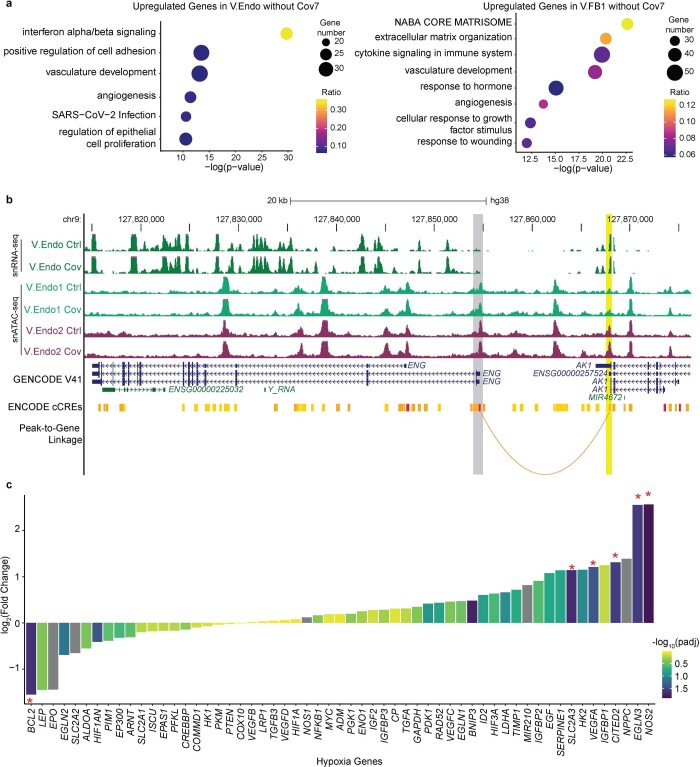
Single-nucleus assays showing angiogenesis dysregulation in fibroblast and endothelial cell types. **a**, GO of upregulated genes in villous endothelial (V.Endo; left) and villous fibroblast 1 (V.FB1; right) cells from the snRNA-seq without the Cov7 (hypertension sample). The colour scale represents the ratio of dysregulated genes to total genes under each GO term; *P* values were calculated using a one-tailed hypergeometric test. **b**, Genome browser screenshot of the region around *ENG*. All pseudo-bulk tracks are displayed as RPM with *y* axes ranging from zero to two. **c**, Expression of hypoxia-related genes from the bulk RNA-seq. The fold change (patient/control) and *P*adj (two-tailed Wald test) were calculated by DESeq2. The genes that are significantly dysregulated (|log2(fold change)| > 1 and *P*adj < 0.05) are marked by a red star.

Placental angiogenesis and remodelling of maternal spiral arteries in the MFI by invasive trophoblasts is vital for successful pregnancy^28^. Abnormal angiogenesis is frequently observed in placental disorders and infections^29,30^. We investigated whether SARS-CoV-2 infection would affect the angiogenesis pathway on a molecular level. We discovered the upregulation of blood vessel development and VEGF signalling genes in vascular endothelial cells and fibroblasts (Fig. 4a,b). Concordantly, increased ATAC-seq peaks in these two cell types were associated with GO terms for vasculogenesis and epithelial cell migration, suggesting epigenetic dysregulation of relevant pathways (Fig. 4c,d). This was also observed by bulk ATAC-seq (Extended Data Fig. 5c). Importantly, sensitivity analyses confirmed that our finding was not confounded by other clinical conditions (Extended Data Fig. 6a). Furthermore, from CellPhoneDB analysis, we found that COVID-19-induced receptor–ligand interactions are significantly associated with the angiogenesis GO term (Extended Data Fig. 5f). These receptor–ligand pairs between endothelial cells, fibroblasts and trophoblasts were further analysed (Fig. 4e). Cell type-specific receptor–ligand pairs relating to angiogenesis—including VEGFA–FLT1 and FGF–FGFR (Fig. 4e), which are known to be critical in angiogenesis during placental development— were significantly upregulated in patients. *VEGFA* was upregulated in fibroblasts and stroma cells (Fig. 4f).

Next, we conducted peak-to-gene linkage analysis using ArchR, which integrates snATAC-seq and snRNA-seq data to predict potential enhancer-gene pairs^31^. We identified a candidate enhancer upstream of the *VEGFA* promoter, coinciding with an ENCODE candidate CRE (cCRE) definition (Fig. 4f). Together with *VEGFA* upregulation, the candidate element gained chromatin accessibility in fibroblasts (Fig. 4f), suggesting that transcriptional dysregulation of angiogenesis pathways may involve epigenetic alterations of associated CREs. We also discovered the upregulation of the *ENG* gene, which encodes the endoglin glycoprotein that is vital for blood vessel development, in villous endothelial cells (Extended Data Fig. 6b). Elevated plasma concentration of soluble ENG is a marker for preeclampsia, which is frequently accompanied by increased circulating VEGF and soluble fms-like tyrosine kinase-1 (sFLT1)^32^. Concomitantly, we detected increased chromatin accessibility at its candidate enhancer in the corresponding cell types (Extended Data Fig. 6b). Furthermore, hypoxia response genes, such as *NOS2* and *EGLN3*, were also upregulated in the patient samples (Extended Data Fig. 6c)^33,34^. Collectively, we found that SARS-CoV-2 induced the dysregulation of angiogenesis genes in vascular endothelial cells and fibroblasts at the MFI, which involved differential activities of putative CREs.

### SARS-CoV-2 induces retrotransposon dysregulation at the MFI

Viral infections, including SARS-CoV-2, are associated with retrotransposon dysregulation^17,35^. However, the exact consequence during pregnancy remains elusive. Therefore, we analysed the transcriptional and epigenomic states of these sequences in patient and control samples. Due to their repetitive natures, next-generation sequencing reads from retrotransposons suffer from poor mappability and are routinely discarded. To circumvent this issue, we utilized our iterative alignment approach termed Subfamily Assignment for Multiple Alignment (SAMA)^36^, which rescues multiple-aligned reads and uniquely anchors them to retrotransposon subfamilies, thereby enabling us to measure transcriptomic and epigenomic changes with higher precision.

**Fig. 5:**
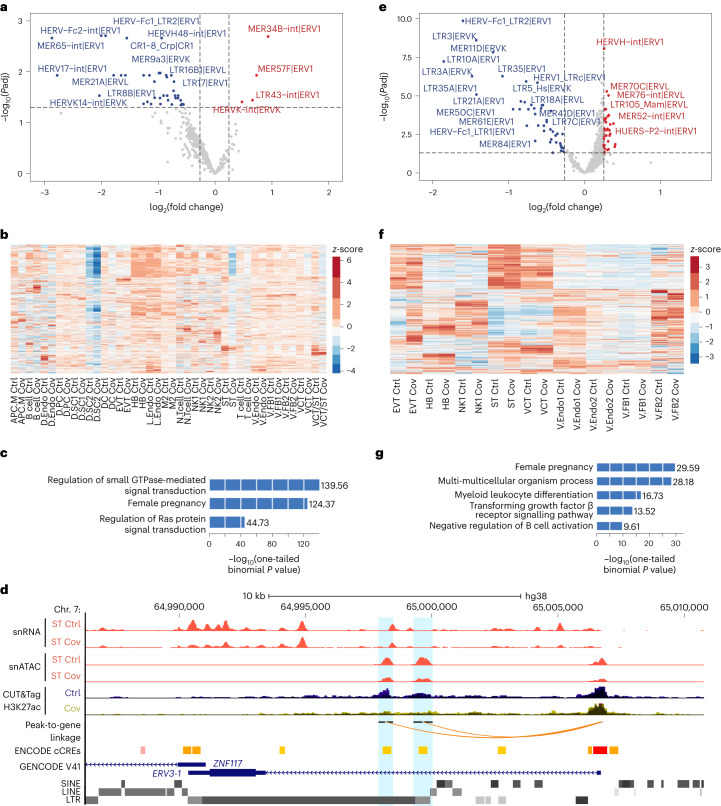
SARS-CoV-2 infection during pregnancy induces dysregulation of retrotransposons. **a**, Dysregulated retrotransposon subfamilies from the bulk RNA-seq. Subfamilies that were significantly upregulated (*P*adj < 0.05 and log_2_(fold change) > 0.25; *n* = 4) or downregulated (*P*adj < 0.05 and log_2_(fold change) < −0.25; *n* = 38) are labelled in red and blue, respectively. **b**, Expression of retrotransposon subfamilies in the 21 cell types from the snRNA-seq. **c**, GREAT analysis of significantly downregulated individual retrotransposons from the bulk RNA-seq analysis. **d**, Genome browser screenshot of *ERV3-1*. The pseudo-bulk tracks from the snRNA-seq and snATAC are displayed as RPM with *y* axes ranging from zero to eight. The CUT&Tag H3K27ac tracks are displayed as aggregated RPM values with *y* axes ranging from zero to four. Blue shadings highlight the candidate enhancers defined by peak-to-gene analysis. **e**, Retrotransposon subfamilies with differential chromatin accessibility from the bulk ATAC-seq. Retrotransposon subfamilies with significantly increased (*P*adj < 0.05 and log_2_(fold change) > 0.25; *n* = 44) or decreased (*P*adj < 0.05 and log_2_(fold change) < −0.25; *n* = 44) accessibility are labelled in red and blue, respectively. **a**,**e**, The negative log_10_(two-tailed Wald test *P*adj) and log_2_(fold change, patient/control) values for each subfamily were calculated by DESeq2. Dashed lines represent the indicated thresholds. **f**, Heatmap showing chromatin accessibility of retrotransposon subfamilies across the nine cell types from the snATAC-seq. **b**,**f**, Each row is a retrotransposon subfamily and the colour scale represents the RPM-calculated row *z*-score. **g**, GREAT analysis of individual retrotransposons with decreased chromatin accessibility from the bulk ATAC-seq. **c**,**g**, The top relevant GO terms are shown. Ctrl, control and Cov, COVID-19; cell-type abbreviations as per Fig. 1b.

**Extended Data Fig. 7:**
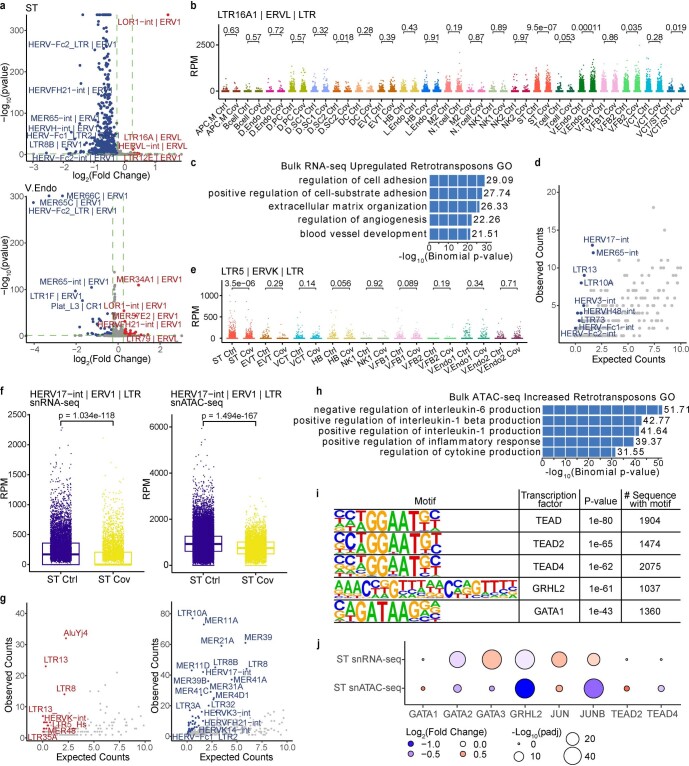
Dysregulation of retrotransposons due to SARS-CoV-2 infection during pregnancy. **a**, Differentially expressed retrotransposon subfamilies in ST (top) and V.Endo (bottom) cells from the snRNA-seq. Upregulated subfamilies (red), *P* < 0.05 and log_2_(fold change) > 0.25, *n* = 6 (ST) and 54 (V.Endo). Downregulated subfamilies (blue), *P* < 0.05 and log_2_(fold change) < −0.25, *n* = 600 (ST) and 57 (V.Endo). *P* value, two-tailed Student’s *t*-test. **b**, Expression of the LTR16A1 subfamily from the snRNA-seq (*n* numbers in Supplementary Table 9). *P* value, two-tailed Student’s *t*-test. **c**, GREAT analysis of upregulated individual retrotransposons from the bulk RNA-seq. *P* value, one-tailed binomial test. **d**, Observed versus expected counts of downregulated individual retrotransposons within subfamilies from the bulk RNA-seq. Subfamilies with observed/expected count > 5 and two-tailed Student’s *t*-test *P* < 0.001 are indicated in blue. **e**, Chromatin accessibility of the LTR5 subfamily from the snATAC-seq (*n* numbers in Supplementary Table 9). *P* value, two-tailed Student’s *t*-test. **f**, Expression (left) and chromatin accessibility (right) of the HERV17-int subfamily from the snRNA-seq and the snATAC-seq, respectively (*n* numbers in Supplementary Table 9). *P* value, two-tailed Student’s *t*-test. **g**, Observed versus expected numbers of individual retrotransposons with increased (left) and decreased (right) chromatin accessibility within subfamilies from the bulk ATAC-seq. Subfamilies with observed/expected count > 5 and two-tailed Student’s *t*-test *P* < 0.001 are shown in red (left) and blue (right). **h**, GREAT analysis of individual retrotransposons with increased chromatin accessibility from the bulk ATAC-seq. *P* value, one-tailed binomial test. **i**, HOMER motif analysis of individual retrotransposons with decreased chromatin accessibility in ST cells from the snATAC-seq. *P* value, one-tailed hypergeometric test. **j**, Transcription factor expression and transcription start site chromatin accessibility called in **i**. *P*adj, one-tailed MAST model from Seurat (snRNA-seq) and one-tailed Poisson test (snATAC-seq). For all boxplots in this figure, the centre and bounds of boxes indicate the median and quartile of all data points, respectively. The minima and maxima of whiskers indicate quartile 1 − 1.5× the interquartile range and quartile 3 + 1.5× the interquartile range, respectively. Source data

From bulk RNA-seq, we discovered four upregulated retrotransposon subfamilies and 38 downregulated subfamilies among patients, including the downregulation of the HERV17-int subfamily from which the *SYNCYTIN-1* gene is derived (Fig. 5a). Interestingly, most dysregulated subfamilies are endogenous retroviruses (ERVs; Fig. 5a). Altered expression of many subfamilies was similarly detected by snRNA-seq (Extended Data Fig. 7a). We also identified cell type-specific dysregulated subfamilies (Fig. 5b). For example, the LTR16A1 subfamily was specifically downregulated in STs, suggesting a cell type-specific regulatory mechanism for these elements (Extended Data Fig. 7b). We then analysed the activities of individual retrotransposons and defined 1,324 upregulated and 7,117 downregulated elements. A Genomic Regions Enrichment of Annotation Tool (GREAT) analysis demonstrated that upregulated elements were associated with the angiogenesis GO term, whereas downregulated elements were related to pregnancy genes (Fig. 5c and Extended Data Fig. 7c). We found several downregulated HERV3-int elements in patients, including an ERV-derived gene (*ERV3-1*), which were specifically downregulated in STs (Extended Data Fig. 7d and Fig. 5d). Intriguingly, peak-to-gene analysis identified two ERV-derived putative enhancers for *ERV3-1*. Concomitantly, the *ERV3-1* gene promoter and the candidate enhancers all lost chromatin accessibility in STs. Moreover, CUT&Tag revealed H3K27ac reduction at these enhancers in patients, which is indicative of reduced *cis*-regulatory activity. Notably, ERV3 class elements are expressed at high levels in the placenta and their decreased expression is linked to pregnancy disorders such as intra-uterine growth restriction^37^. Our findings suggest that pregnancy-related ERVs are dysregulated in the MFI in patients with COVID-19, which is associated with epigenetic reprogramming of ERV-derived CREs.

We further investigated retrotransposon-derived CREs in patients by assessing their chromatin accessibility. Utilizing SAMA, we identified retrotransposon subfamilies with varied bulk ATAC-seq signals in patients (Fig. 5e). Furthermore, snATAC-seq uncovered cell type-specific dysregulation, which was obfuscated in the bulk data (Fig. 5f). We found subfamilies, including HERV-K/LTR5, that significantly gained chromatin accessibility specifically in STs (Extended Data Fig. 7e). In addition, downregulated subfamilies identified in snRNA-seq showed decreased chromatin accessibility in corresponding cell types. For instance, HERV17-int showed both transcriptional downregulation and chromatin accessibility loss in STs (Extended Data Fig. 7f). Focusing on elements with altered snATAC-seq signal (Extended Data Fig. 7g), we discovered association with the inflammatory response GO term (Extended Data Fig. 7h). Interestingly, retrotransposons with decreased chromatin accessibility were also associated with the female pregnancy GO term (Fig. 5g). From snATAC-seq, elements with decreased chromatin accessibility in STs are enriched with important placental transcription factor binding motifs, including GATA2 and GRHL2 (Extended Data Fig. 7i). Notably, the expression levels of *GATA2* and *GRHL2* were also reduced in STs, consistent with loss of chromatin accessibility at their promoters (Extended Data Fig. 7j). Together, these results demonstrate changes in retrotransposon activities at the MFI following SARS-CoV-2 infection, which may influence important processes in pregnancy.

### *PSG* downregulation coincides with reduced LTR8B activity

**Fig. 6:**
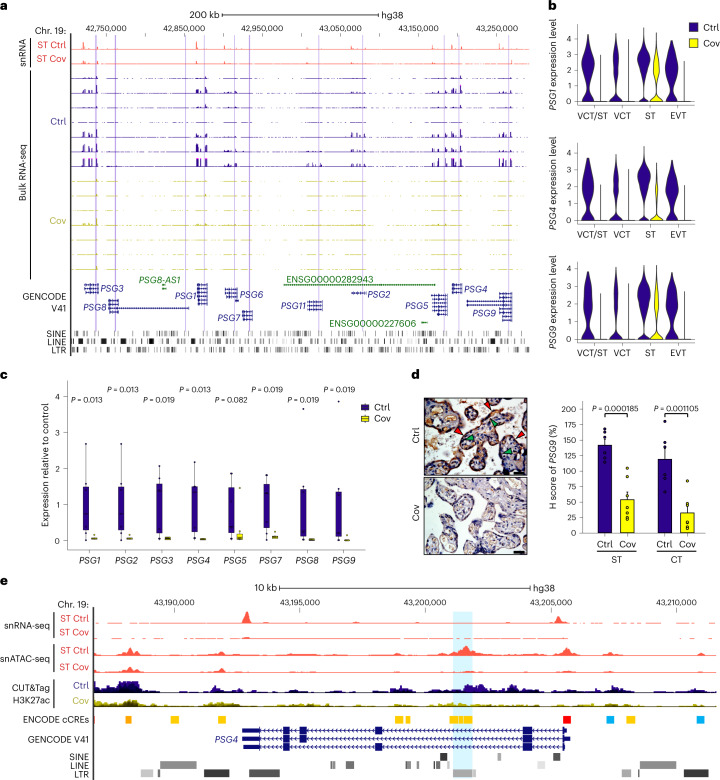
Reduction of pregnancy gene expression is associated with loss of chromatin accessibility at intronic LTR elements. **a**, Genome browser screenshot of the *PSG* gene cluster in the STs from the snRNA-seq and bulk RNA-seq. The purple shading highlights LTR8B elements within each *PSG* gene. The pseudo-bulk tracks from the snRNA-seq and bulk RNA-seq tracks are displayed as RPM with *y* axes ranging from 0 to 300 and 0 to 100, respectively. **b**, Average expression levels, calculated by Seurat, of the *PSG1* (top), *PSG4* (middle) and *PSG9* genes (bottom) in trophoblast cell types from the snRNA-seq in patient and control samples. **c**, Expression levels, determined using RT–qPCR, of eight *PSG* genes in patient and control samples. Expression was normalized to *TBP*. Each dot represents a sample (*n* = 7 for both patient and control); *P* values were calculated using a one-tailed Wilcoxon test. The centre and bounds of boxes indicate the median and quartile of all data points, respectively. The minima and maxima of whiskers indicate quartile 1 − 1.5× the interquartile range and quartile 3 + 1.5× the interquartile range, respectively. **d**, Immunohistochemistry staining (left) and H scores (right) of PSG9 protein in patient and control samples. Red arrowheads indicate STs and green arrowheads indicate cytotrophoblasts. Scale bars, 50 µm. Each dot in the bar chart represents a sample (*n* = 7 for patient and *n* = 6 for control). Data are presented as the mean values with error bars showing the s.e.m.; *P* values were calculated using a two-tailed Student’s *t*-test. **e**, Genome browser screenshot showing the *PSG4* gene and its intronic LTR8B element, highlighted by the blue shading. The pseudo-bulk tracks from the snRNA-seq and snATAC-seq are displayed as RPM. The H3K27ac CUT&Tag tracks are displayed as aggregated RPM. The *y* axes range from 0 to 60 for the snRNA-seq, 0 to 4 for the snATAC-seq and 0 to 2 for the CUT&Tag. Ctrl, control and Cov, COVID-19; cell-type abbreviations as per Fig.  1b. Source data

**Extended Data Fig. 8:**
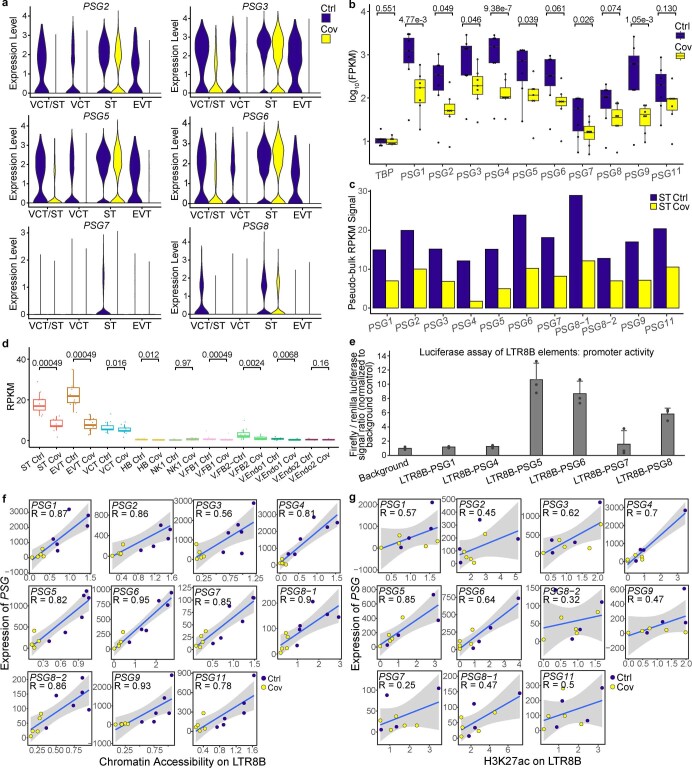
Downregulation of the expression of *PSG* genes due to SARS-CoV-2 infection during pregnancy. **a**, Expression of *PSG2* (top left), *PSG3* (top right), *PSG5* (middle left), *PSG6* (middle right), *PSG7* (bottom left) and *PSG8* (bottom right) in the trophoblast cell types from the snRNA-seq calculated by Seurat. **b**, Expression of *PSG* genes from the bulk RNA-seq. Each dot represents a sample (*n* = 7 for both patient and control) and the *P*adj (two-tailed Wald test) was calculated by DESeq2. **c**, Chromatin accessibility signal of each intronic LTR8B within a *PSG* gene in the ST cell type from the snATAC-seq. **d**, Chromatin accessibility signal of intronic LTR8B elements within *PSG* genes in all nine cell types from the snATAC-seq. Each dot represents one LTR8B element (*n* = 11); *P* values were calculated using a one-tailed Wilcoxon test. **e**, Luciferase assay for promoter activity of individual intronic LTR8B elements within different *PSG* genes. Data are presented as mean values with error bars showing the s.d. and each dot representing an independent experiment (*n* = 3). **f**,**g**, Correlations between bulk RNA-seq expression levels of *PSG*s and the bulk ATAC-seq signals (**f**) as well as the H3K27ac CUT&Tag signals (**g**) of corresponding intronic LTR8B elements. The *y* axes represent the FPKM signal from the bulk RNA-seq and the *x* axes represent the RPKM signal from the respective assays. The error bands in grey represent a 95% confidence level interval for predictions from a linear model; Spearman’s correlation was used to calculate the *R* value. For all boxplots in this figure, the centre and bounds of boxes indicate the median and quartile of all data points, respectively. The minima and maxima of whiskers indicate quartile 1 − 1.5× the interquartile range and quartile 3 + 1.5× the interquartile range, respectively. Source data

We observed a downregulation of pregnancy-related genes, including *PSG*, at the MFI of the pregnant patients with SARS-CoV-2 infection (Fig. 2a). The human genome contains ten bona fide *PSG* genes, *PSG1*–*9* and *PSG11*, which are clustered on chromosome 19q13 (ref. ^38^). They are expressed at high levels during pregnancy and are involved in the processes of immune modulation and angiogenesis^38^. Reduced PSG protein levels in serum are associated with adverse pregnancy outcomes, including pregnancy loss and preeclampsia^39,40^. However, their precise regulatory mechanism remains poorly understood. We found that seven *PSG* genes were downregulated in the STs of patients infected with COVID-19 (Fig. 6a,b and Extended Data Fig. 8a,b). We validated their transcriptional and protein level changes using RT–qPCR and immunohistochemical staining, respectively (Fig. 6c,d). Intriguingly, all *PSG* genes harbour intronic LTR8B elements, which have high open chromatin signals and H3K27ac enrichment in control samples (Fig. 6a,e). We found the downregulation of *PSG* genes occurred concomitantly with decreased chromatin accessibility and H3K27ac levels at intronic LTR8B elements (Fig. 6e and Extended Data Fig. 8c). Moreover, these retrotransposons showed trophoblast-specific open chromatin states (Extended Data Fig. 8d). We postulate that these elements serve as trophoblast-specific CREs for the *PSG* genes.

### LTR8B elements function as enhancers in placental cells

**Fig. 7:**
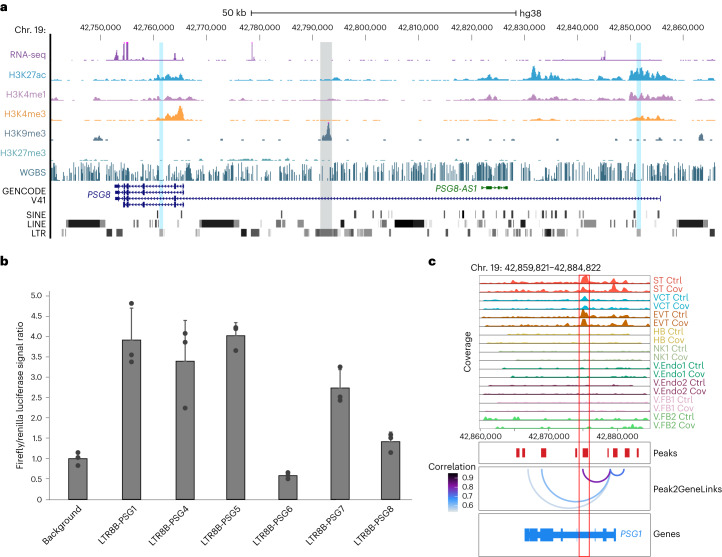
LTR8B elements function as enhancers in trophoblast cells. **a**, Genome browser screenshot showing the transcriptional and epigenetic states of the *PSG8* gene in primary STs. The displayed datasets were obtained from the International Human Epigenome Consortium data repository^41,42^. The intronic ERV1 element is shaded in grey and the LTR8B elements within *PSG8* are shaded in blue. All tracks are displayed as RPM, with *y* axes ranging from 0 to 200 for RNA-seq, 0 to 100 for H3K27ac ChIP–seq, 0 to 150 for H3K4me1 ChIP–seq, 0 to 250 for H3K4me3 ChIP–seq, 0 to 20 for H3K9me3 ChIP–seq, 0 to 40 for H3K27me3 ChIP–seq and 0 to 1 for whole-genome bisulphite sequencing (WGBS). **b**, Luciferase assay enhancer activity of individual intronic LTR8B elements within different *PSG* genes normalized to the background control. Each dot represents an independent experiment (*n* = 3) and data are presented as mean values with error bars showing the s.d. **c**, Peak-to-gene linkage analysis indicating a potential interaction between the *PSG1* promoter and its intronic LTR8B (highlighted by the red box). Tracks are displayed as normalized pseudo-bulk coverage signals of each cell type from the snATAC-seq ranging from 0 to 0.25. Ctrl, control and Cov, COVID-19; cell-type abbreviations as per Fig. 1b. Source data

We next investigated the enhancer potentials of the intronic LTR8B elements in regulating the *PSG* genes. Publicly available RNA-seq and chromatin immunoprecipitation-sequencing (ChIP–seq) datasets from primary placenta cell types were analysed^41,42^. We found that high expression of *PSG* genes in STs was accompanied by enrichment of active enhancer histone modification signatures (high H3K27ac and H3K4me1, and low H3K4me3) at the intronic LTR8B elements (Fig. 7a). As expected, repressive modifications—such as H3K9me3, H3K27me3 and DNA methylation—were depleted at these loci (Fig. 7a). Notably, another ERV1 cluster in the same intron of *PSG8* was marked by H3K9me3, suggesting that the activation of these LTR8Bs did not result from positional effects (Fig. 7a). We validated the enhancer functionality of LTR8Bs using a luciferase assay in which most elements demonstrated strong enhancer activities (Fig. 7b and Extended Data Fig. 8e).

**Fig. 8:**
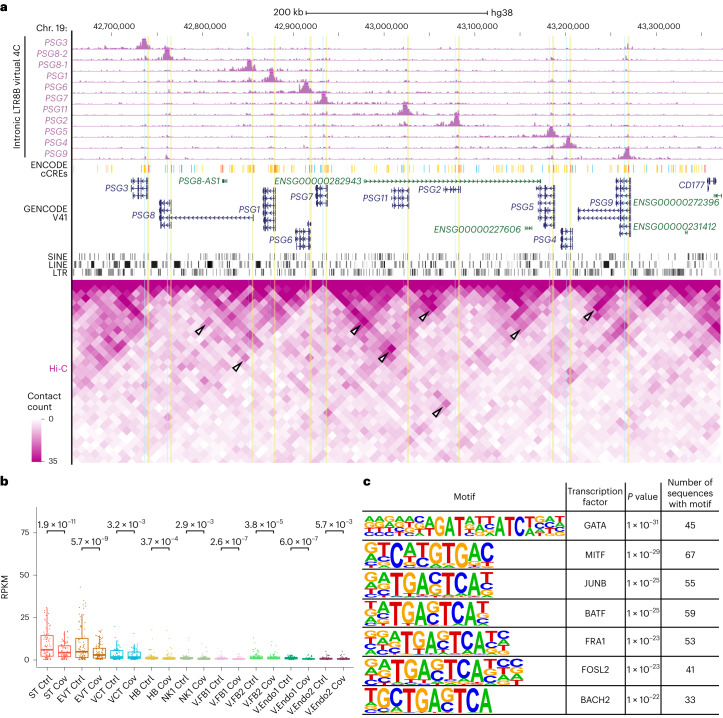
Intronic LTR8B elements interact with *PSG* gene promoters. **a**, Screenshot of the virtual 4C results (top) and Hi-C interaction frequency (bottom) from TSCs at the *PSG* cluster showing the interaction of *PSG* gene promoters and intronic LTR8B elements. The virtual 4C shows interaction frequencies using each intronic LTR8B element as an anchor. The intronic LTR8B elements and the promoters of *PSG* genes are highlighted with blue and yellow shading, respectively. The tracks are displayed as RPKM, with *y* axes ranging from 0 to 20,000. The white arrowheads in the Hi-C heatmap point to interactions within the *PSG* cluster, indicating a high interaction frequency between intronic LTR8B and the promoters of *PSG* genes. **b**, Pseudo-bulk RPKM signal of all active LTR8B elements defined by co-accessibility analysis (*n* = 83) in the nine cell types from the snATAC-seq. Each dot represents one active LTR8B element; *P* values were calculated using a one-tailed paired Student’s *t*-test. The centre and bounds of the boxes indicate the median and quartile of all data points, respectively. The minima and maxima of whiskers indicate quartile 1 − 1.5× the interquartile range and quartile 3 + 1.5× the interquartile range, respectively. **c**, HOMER motif analysis of active LTR8B elements defined by the co-accessibility analysis (*n* = 83). *P* values were calculated using a one-tailed hypergeometric test. Ctrl, control and Cov, COVID-19; cell-type abbreviations as per Fig. 1b. Source data

**Extended Data Fig. 9:**
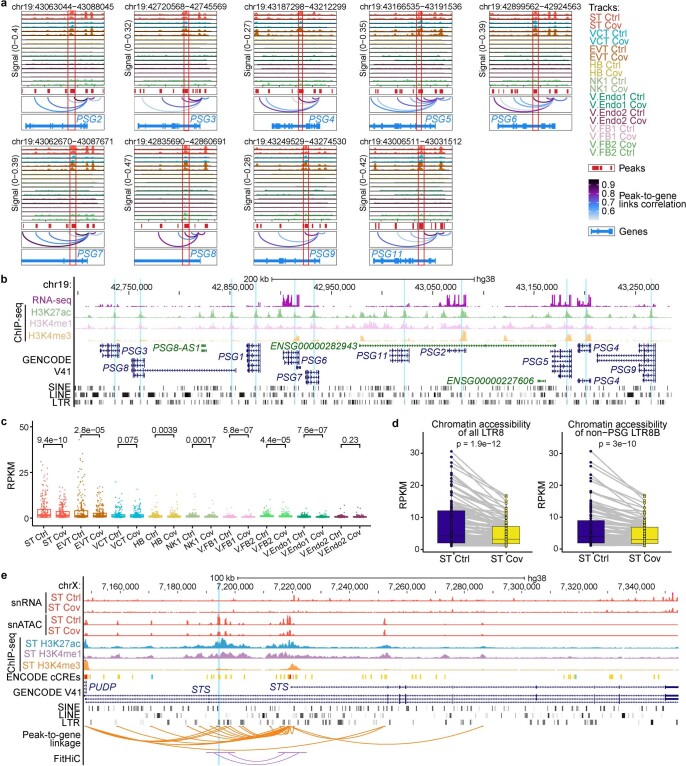
Decreased expression of *PSG* genes is associated with reduced intronic LTR8B chromatin accessibility. **a**, Peak-to-gene linkage analysis of *PSG* genes and their corresponding intronic LTR8B elements (red boxes). Tracks are displayed as pseudo-bulk signals of each cell type from the snATAC-seq and the colour scale represents the correlation value. **b**, Genome browser screenshot of the *PSG* gene cluster with expression and histone modifications tracks of TSCs. The blue shadings highlight the intronic LTR8B elements within *PSG* genes. All tracks are displayed as RPM, *y* axes are range from 0 to 2 for RNA-seq, 0 to 80 for H3K27ac, 0 to 40 for H3K4me1 and 0 to 80 for H3K4me3. **c**, Chromatin accessibility of LTR8B individual elements in all cell types from the snATAC-seq. Each dot represents an LTR8B element; *P* values were calculated using a one-tailed Wilcoxon test. Elements were filtered for RPKM > 1 in at least one cell type and length > 400 bp (*n* = 242). **d**, Chromatin accessibility of all LTR8B individual elements (left; *n* = 125) and LTR8B elements outside the *PSG* cluster (right; *n* = 114). Each dot represents an LTR8B element; *P* values were calculated using a one-tailed Wilcoxon test. LTR8B elements were filtered by RPKM > 1 in either control or patient sample and length > 400 bp. **e**, Genome browser screenshot of the *STS* gene promoter region. The blue shading highlights an LTR8B element. All pseudo-bulk tracks from the snRNA-seq, snATAC-seq and ChIP–seq tracks from the IHEC data repository are displayed in RPM. Significant interactions (one-tailed binomial test *P*adj < 0.05) predicted by Fit-Hi-C are displayed by purple arcs. The *y* axes are range from 0 to 15 for snRNA-seq, 0 to 6 for snATAC-seq, 0 to 200 for H3K27ac, 0 to 200 for H3K4me1 and 0 to 500 for H3K4me3. For all boxplots in this figure, the centre and bounds of boxes indicate the median and quartile of all data points, respectively. The minima and maxima of whiskers indicate quartile 1 − 1.5× the interquartile range and quartile 3 + 1.5× the interquartile range, respectively.

We found that the degree of chromatin accessibility and H3K27ac enrichment at the LTR8Bs are highly correlated with the expression of corresponding *PSG* genes (Extended Data Fig. 8f,g), providing support for their CRE identity. Moreover, peak-to-gene linkage analysis from snATAC-seq identified strong linkages between *PSG* promoters and their intronic LTR8B elements (Fig. 7c and Extended Data Fig. 9a). To elucidate the precise targets of LTR8B enhancers, we performed Hi-C analysis of the human expanded potential stem cell-derived trophoblast stem cells (TSCs)^43^, which had high expression of most *PSG* genes and enrichment of active enhancer marks at the intronic LTR8Bs (Extended Data Fig. 9b). Strong three-dimensional interactions between the intronic LTR8B elements and multiple *PSG* gene promoters were defined (Fig. 8a), suggesting that these retrotransposons formed a regulatory network with *PSG* genes.

To determine whether LTR8B elements broadly functioned as placental enhancers and whether they were commonly affected by SARS-CoV-2, we interrogated other elements within the subfamily. The LTR8B subfamily possessed high chromatin accessibility in trophoblasts compared with other cell types, which was significantly reduced in the COVID-19 samples (Extended Data Fig. 9c,d). We defined 83 LTR8B elements as putative genic enhancers from peak-to-gene linkage analysis, which had high chromatin accessibility in trophoblasts (Fig. 8b). Following SARS-CoV-2 infection, a substantial loss of ATAC-seq signal was detected in all cell types, with the most significant change in STs (Fig. 8b). Moreover, these active elements were enriched with placental developmental transcription factor motifs, including GATA, JUNB and FOSL2 (Fig. 8c). For example, an LTR8B element was predicted to be a potential enhancer for the downregulated *STS* gene in STs (Extended Data Fig. 9e). This element was enriched with H3K27ac and H3K4me1 in primary ST cells. Concordant with *STS* downregulation, the LTR8B element lost chromatin accessibility in the COVID-19 samples. Furthermore, Hi-C showed three-dimensional chromatin interactions between the LTR8B element and the *STS* promoter in TSCs. Together, our results indicate that a subset of LTR8B retrotransposons function as enhancers in regulating placental genes and SARS-CoV-2 infection is linked to decreased activity of both these CREs and their targets.

### COVID-19 infection during pregnancy induces epigenetic reprogramming of LTR8Bs

**Extended Data Fig. 10:**
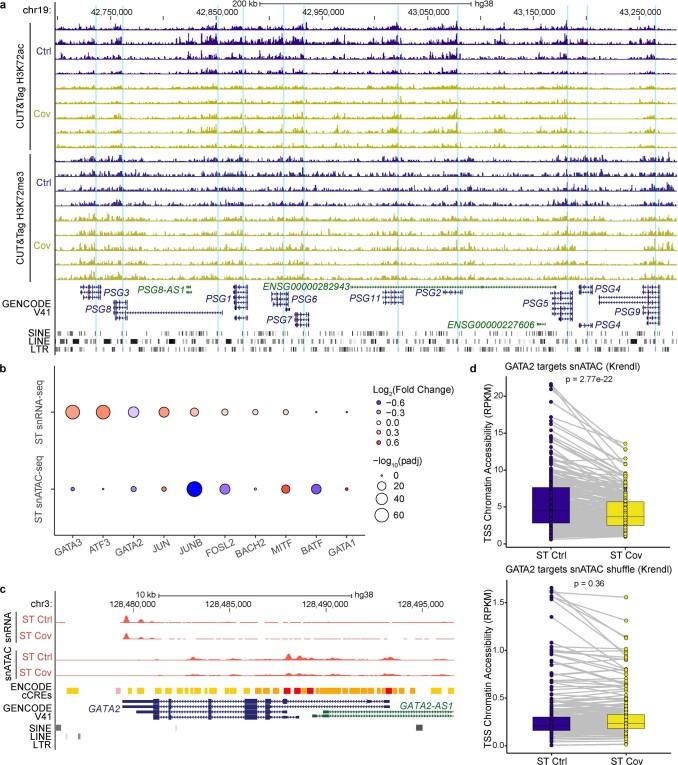
Epigenetic regulation of *PSG* genes in trophoblasts and decreased expression of GATA2 in patient samples. **a**, Genome browser screenshot of the *PSG* gene cluster showing the H3K27ac and H3K27me3 CUT&Tag signals. All tracks are displayed as RPM, with *y* axes ranging from zero to three for H3K27ac and zero to two for H3K27me3. The blue shadings highlight the intronic LTR8B elements within *PSG* genes. **b**, Expression and chromatin accessibility of transcription factors in the ST cell type called in Fig. 8c. The *P*adj was calculated using the one-tailed MAST model from Seurat for snRNA-seq and a one-tailed Poisson test for snATAC-seq. **c**, Genome browser screenshot showing expression and chromatin accessibility of *GATA2* in the ST cell type from the snRNA-seq and the snATAC-seq. All tracks are displayed as RPM, with *y* axes ranging from 0 to 40 for snRNA-seq and 0 to 5 for snATAC-seq. **d**, Chromatin accessibility of *GATA2*-targeted genes transcriptional start site regions (top; *n* = 245) and number-matched shuffle control (bottom) between patient and control samples in the ST cell type from the snATAC-seq. Each dot represents a *GATA2-*targeted gene; *P* values were calculated using a one-tailed Wilcoxon test. The centre and bounds of boxes indicate the median and quartile of all data points, respectively. The minima and maxima of whiskers indicate quartile 1 − 1.5× the interquartile range and quartile 3 + 1.5× the interquartile range, respectively. Source data

Given the transcriptional and chromatin state changes at the LTR8Bs and *PSG* genes, we further aimed to delineate the underlying molecular mechanism. We analysed the H3K27me3 and H3K27ac CUT&Tag data from control and patient samples. Although no significant change in H3K27me3 was detected, we found reduced H3K27ac at LTR8Bs in the patients with COVID-19 infection (Extended Data Fig. 10a), which correlated with *PSG* expression (Extended Data Figs. 8g and 10a). These results revealed that the cell type-specific downregulation of LTR8B elements was coupled with H3K27ac loss and was probably unrelated to H3K27me3 enrichment.

Reduced levels of transcription factor binding to CREs may also explain the downregulation of *PSG* genes. The motif for GATA was the most significantly enriched transcription factor motif in active LTR8B elements (Fig. 8c). GATA proteins are master regulators in placenta development^44^. Interestingly, we found that *GATA2* expression and promoter chromatin accessibility were significantly reduced in the STs of the patients with COVID-19 (Extended Data Fig. 10b,c). Furthermore, known target genes of GATA2 also exhibited loss of ATAC-seq signals (Extended Data Fig. 10d)^45^. Together, we surmised that LTR8B-derived enhancers, which are normally accessible and enriched with H3K27ac in STs, regulate the *PSG* genes via GATA2 binding. However, these retrotransposons lose activity following SARS-CoV-2 infection, which could be due to aberrant epigenetic regulation and/or reduction of GATA2 recruitment.

## Discussion

Throughout the COVID-19 pandemic, hospitals have seen increasing numbers of pregnant patients who are infected with SARS-CoV-2. Although mounting evidence suggests that patients face increased risks of adverse pregnancy outcomes, the underlying molecular mechanism remains unclear. In this study the participating patients were diagnosed with COVID-19 during late pregnancy. To discriminate the effect of maternal immune response from a direct viral infection, we confirmed that the patient MFI samples were free from the virus at the time of analysis. Nonetheless, immune and angiogenesis dysregulation was observed at the MFI of patients. This could expose the fetus to inflammation, hypoxia and oxidative stress, which can substantially impact the fetal epigenome and developmental process. Our findings suggest that patients with COVID-19 have a higher potential for placenta-related complications, and specific care and management should be instigated.

The complex immunomodulation at the MFI is important for successful pregnancies. Aberrant interferon expression is known to be a common cause of pregnancy disorders^46^. Here we detected upregulation of interferon-related genes across different cell types. IFITM proteins were demonstrated to inhibit syncytin-mediated ST formation in mouse models, which results in placental abnormalities^47^. The process of ST formation occurs from implantation to full term^48^. Therefore, the overexpression of *IFITM* genes in late pregnancy can still potentially be detrimental. Furthermore, type I interferon response towards SARS-CoV-2 can cause lung damage by cGAS–STING activation^49^, which may similarly apply to placental endothelial cells.

Abnormal angiogenesis is found in a variety of pregnancy complications; however, their regulatory pathways in individuals with COVID-19 have not been fully explored. We found that angiogenesis genes are upregulated in endothelial cells and fibroblasts, concomitant with altered chromatin accessibility at CREs. We discovered putative enhancers upstream of *VEGFA* and *ENG*. Both genes and their CREs showed higher expression and chromatin accessibility in patients with COVID-19, respectively. *VEGFA* and *ENG* are known to be vital to early placental vascular development and are transcriptionally dysregulated in patients with preeclampsia^32^. The dysregulation of these genes may be associated with endothelial dysfunction in patients infected with COVID-19.

We also discovered retrotransposon dysregulation in patient samples. Strikingly, hundreds of elements were differentially expressed and/or had altered chromatin states. Downregulated elements were associated with pregnancy genes and enriched with motifs for important transcription factors, indicating involvement in normal placental functions. For instance, a subset of LTR8B elements serve as enhancers for the *PSG* genes. *PSG* genes encode glycoproteins that have immunoregulatory, pro-angiogenic and anti-platelet functions. Their disruption is associated with pregnancy complications including preeclampsia^38,40^. We found that the deregulation of LTR8B-derived enhancers is potentially responsible for *PSG* downregulation in the STs of patients with COVID-19. Hi-C data confirmed the strong higher-order chromatin interactions between the retrotransposons and multiple *PSG* promoters, indicating potential *PSG* regulatory modules. The H3K27ac enrichment at LTR8B elements is correlated with *PSG* expression levels. These downregulated elements are enriched with binding motifs for GATA2, which is also transcriptionally downregulated in patients. We propose that reduced H3K27ac at these retrotransposon-derived enhancers and downregulation of *GATA2* point to reduced enhancer activities that results in decreased *PSG* expression in SARS-CoV-2 infection. Such changes potentially cause impaired immunoregulation and angiogenesis at the MFI.

Collectively, we generated extensive multi-omic datasets of the MFI from patients infected with SARS-CoV-2 and control individuals. Our findings uncovered the critical role of epigenetic regulation and defined retrotransposon-derived enhancers associated with the altered expression of important pregnancy genes. Further studies investigating whether new SARS-CoV-2 variants could induce similar immune activation and angiogenesis dysregulation should be carried out.

## Methods

### Patient consent and sample collection

This study complies with all relevant ethical regulations. Approval was obtained from the Joint Chinese University of Hong Kong—New Territories East Cluster Clinical Research Ethics Committee (CREC ref. no. 2020.210) and Hong Kong University of Science and Technology (ref. no. HREP2021-0100 and HREC548).

Our case-control study included seven consecutively sampled pregnant patients who tested positive for SARS-CoV-2 by RT–qPCR (*C*_t_ ≤ 35) of deep throat saliva or nasopharyngeal swabs beyond 24 weeks of gestation and seven uninfected women. All participants gave written informed consent to participate in the study and for the results to be published. No compensation was provided to participants. Blood and cord blood were collected from the patients immediately after delivery and serum antibodies to SARS-CoV-2 were analysed. Qualitative detection of the anti-SARS-CoV-2 immunoglobulin G (IgG) to the SARS-CoV-2 nucleocapsid protein (N-protein) was performed using an Elecsys Anti-SARS-CoV-2 assay (Roche) on a Cobas e411 analyser.

Tissues from the MFI were collected within 2 h after delivery as previously described^50^. All procedures were performed in a certified Class II Biosafety cabinet. For diagnostic RT–qPCR, RNA was extracted from tissues taken midway between the umbilical cord insertion site and the edge of the placental disk. The tissue was placed with the basal plate on the uppermost orientation and several random sampling sites without frank pathology were identified. Visible blood vessels and blood clots were removed. The collected tissues were gently rinsed in sterile cold PBS to minimize maternal-blood carryover. All tissues were collected by the same person to avoid interpersonal variation in the collection practice. The samples were immersed in 10 ml RNAlater (Ambion) for 24 h at ambient temperature and stored in a freezer at −80 °C until RNA extraction.

For immunohistochemical assays, the washed samples were fixed with 4% paraformaldehyde solution for 24–48 h and dehydrated to enable embedding with paraffin^51^.

For other assays, the washed samples were snap-frozen in liquid nitrogen and stored in a −80 °C freezer. The frozen specimens were pulverized using a mortar and pestle with added liquid nitrogen. The resulting powdered samples were stored in a freezer at −80 °C until further experiments.

### Detection of SARS-CoV-2 in patient samples

Three pieces of tissue were used from each case and amplified in duplicate. Total RNA was extracted using an RNeasy mini kit (Qiagen). The detection of SARS-CoV-2 RNA was performed with the FDA-authorized CDC 2019-novel coronavirus (2019 nCoV) real-time RT-PCR diagnostic panel (EUA 200001). The nucleocapsid genes (both *N1* and *N2*) were assayed with human *RNase P* as an endogenous reference control. The DNA sequence of the SARS-CoV-2 *N* gene (10006625, IDT) was used as the positive control. A set of in vitro-synthesized RNA transcripts including three quantification positive controls (1,000, 100 and 10 copies genome equivalent) were also assayed.

### Immunohistochemistry

Formalin‐fixed paraffin-embedded tissue blocks were sectioned to a thickness of 5 μm for standard haematoxylin and eosin as well as specific immunohistochemical staining. The slides were deparaffinized with xylene and rehydrated with ethanol. For the immunohistochemical staining, the slides were treated with 3% hydrogen peroxide, followed by heating in antigen retrieval buffer (pH 6.0; Abcam), incubation with Protein Block (Abcam) and overnight incubation at 4 °C with primary antibodies to FLNB (ab282106, Abcam), PAPPA (ab174314, Abcam) and PSG9 (AP53483PU-N, Origene). After washing with 0.1% TBS in Tween‑20, the slides were incubated with the secondary antibody horseradish peroxidase (HRP)-conjugated mouse anti-rabbit (Sigma-Aldrich), stained with the substrate 3,3′-diaminobenzidine (EnVision) and counterstained with haematoxylin (Sigma-Aldrich).

### Human TSC culture

The TSCs were a gift from P. Liu (School of Biomedical Sciences, The University of Hong Kong)^43^. The TSCs were cultured as previously described^52^ with minor modifications. Briefly, the cells were maintained in TSC medium (DMEM/F12 supplemented with 0.1 mM 2-mercaptoethanol, 0.2% fetal bovine serum, 0.5% penicillin–streptomycin, 0.3% BSA, 1% ITS-X supplement, 150 µM l-ascorbic acid, 50 ng ml^−1^ EGF, 2 mM CHIR99021, 0.5 mM A83-01, 1 mM SB431542, 0.8 mM VPA and 5 mM Y27632). Tissue culture plates were coated with 5 µg ml^−1^ collagen IV (Corning) at 37 °C for 1 h. The cells were cultured at 37 °C with 5% CO_2_. TSCs at 20–30 passages were harvested for analyses.

### Bulk RNA-seq and RT–qPCR

Total RNA from 10–20 mg of pulverized tissue or 1 – 2 × 10^6^ pelleted TSCs was extracted with TRIzol reagent (Invitrogen) according to the manufacturer’s manual. The extracted total RNA was used for generating bulk RNA-seq libraries and RT–qPCR. For RNA-seq, 1 µg total RNA underwent ribosomal RNA depletion using a Ribo-off rRNA depletion kit (Vazyme), followed by RNA-seq library preparation using a QIAseq stranded total RNA library kit (Qiagen), as described in the manufacturer’s manual, and sequenced on an Illumina NextSeq 500 platform.

For RT–qPCR analyses of DEGs, which were performed separately from the SARS-CoV-2 diagnostic RT–qPCR, 1 µg total RNA was treated with DNase I (NEB) and purified with RNAClean XP beads (Beckman Coulter). First-strand synthesis was performed using a Superscript III reverse transcription system (Thermo Fisher Scientific) according to the manufacturer’s manual. The complementary DNA was analysed by qPCR on a LightCycler 480 Instrument II. The RT–qPCR primers are listed in Supplementary Table 7.

### Bulk ATAC-seq library preparation

The ATAC-seq protocol was adopted from Buenrostro et al.^53^, with minor modifications. Briefly, 10–20 mg pulverized tissue was resuspended in 1 ml nuclei permeabilization buffer (5% BSA, 0.2% NP-40, 1 mM dithiothreitol and 1×protease inhibitors in PBS), incubated for 10 min at 4 °C with rotation and filtered through a 40-µm Cell Strainer (Corning). The nuclei were pelleted at 500*g* and 4 °C for 5 min and resuspended in 50 μl of chilled tagmentation buffer (Vazyme). Nuclei in suspension were counted using a haemocytometer and the concentration was adjusted to 2,000–5,000 nuclei µl^−1^. Vazyme V50 Tn5 transposase (0.5 µl) was added to 9.5 µl of the nuclei suspension, followed by incubation in a thermomixer at 37 °C with mixing at 500 r.p.m. for 30 min. The tagged DNA was amplified using KAPA HiFi hotstart ready mix (Roche) for 5–10 PCR cycles, followed by size selection with Ampure XP beads (Beckman Coulter) and sequenced on an Illumina NextSeq 500 platform.

### CUT&Tag library preparation

CUT&Tag libraries were built based on a published protocol^54^. Briefly, the pulverized tissues were washed once with wash buffer (20 mM HEPES pH 7.5, 150 mM NaCl, 0.5 mM spermidine and 1×protease inhibitor cocktail). Activated ConA beads (30 µl) were added to about 150,000 nuclei, followed by resuspension in 150 µl antibody buffer and division into three tubes. Primary antibody (1 µl; anti-H3K27me3 Active motif 39155, anti-H3K27ac Active motif 39133 or rabbit IgG antibody Sigma-Aldrich, I5006) was added to each tube and the samples were incubated overnight at 4 °C. The next day, the buffer was changed to Dig-Wash buffer (20 mM HEPES pH 7.5, 150 mM NaCl, 0.5 mM spermidine, 1×protease inhibitor cocktail and 0.05% digitonin) containing 1:100 secondary antibody (donkey anti-rabbit IgG; Abcam, ab6701) and incubated at room temperature for 1 h. The beads were washed with Dig-Wash buffer and resuspended in 100 µl Dig-300 buffer (0.05% digitonin, 20 mM HEPES pH 7.5, 300 mM NaCl, 0.5 mM spermidine and 1×protease inhibitor cocktail) containing 1:40 pA-Tn5 adaptor complex. These tubes were incubated at room temperature for 1 h, washed with Dig-300 buffer, resuspended in 300 µl Tagmentation buffer and incubated at 37 °C for 1 h. The tagged DNA was purified using a Qiagen MinElute PCR purification kit. The libraries were PCR amplified for 13 cycles using NEBNext HiFi 2× PCR master mix, purified with Ampure XP beads and sequenced on an Illumina NextSeq 500 platform.

### Preparation of snRNA-seq and snATAC-seq libraries

Pulverized tissue (10–20 mg) was resuspended in 1 ml nuclei permeabilization buffer, incubated with rotation for 10 min at 4 °C and filtered through a 40-µm Cell Strainer (Corning). The nuclei were pelleted at 500*g* and 4 °C for 5 min and used for snRNA-seq library preparation using a Chromium Next GEM Single Cell 3ʹ V3.1 kit and snATAC library preparation using a Chromium next GEM single cell ATAC v1.1 kit. The libraries were converted for sequencing on the MGI sequencing platform using an MGIEasy universal library conversion kit. The converted libraries were sequenced on the MGISEQ-2000RS platform.

### Hi-C library preparation

The TSC Hi-C library was generated using an Arima-Hi-C kit according to the manufacturer’s protocol. The library was converted for sequencing on the MGI sequencing platform with an MGIEasy universal library conversion kit and sequenced on the MGISEQ-2000RS platform.

### Micro-ChIP–seq library preparation

TSC micro-ChIP–seq libraries were prepared as described previously^55^, with minor modifications. Briefly, 5 × 10^5^ crosslinked cells were resuspended in lysis buffer and sonicated using a Covaris S220 sonicator continuously for 400 s at 175 W. Fragmented chromatin was added to Protein A Dynabeads (Thermo Fisher Scientific) bound to antibodies to H3K27ac (AM39133, Active Motif), H3K4me3 (AM39915, Active Motif) or H3K4me1 (AM91289, Active Motif). The mixtures were incubated at 4 °C for 40 h with rotation. The captured chromatin was washed four times with RIPA buffer. Reverse crosslinking was performed by incubation at 68 °C for 4 h with proteinase K. The eluted DNA was purified using a Qiagen MinElute PCR purification kit. Libraries were prepared using a KAPA HyperPrep Kit (Roche) and sequenced on an Illumina NextSeq 500 platform.

### Luciferase assays

The *PSG* intronic LTR8B elements or *GFP* sequences (negative control) were cloned into the pGL3-promoter and pGL3-enhancer vectors. The primers used for amplification of target sequences are listed in Supplementary Table 7. Two days before transfection, TSCs were seeded at 4 × 10^4^ cells per well in 24-well plates. The cells were co-transfected with the *Renilla* luciferase vector and pGL3 vector containing an LTR8B element or GFP sequence with Lipofectamine 3000 transfection reagent (Thermo Fisher). Luciferase activity was measured using Dual-luciferase reporter assay system reagents (Promega, E1960) according to the manufacturer’s protocol with a Spectronic Genesys 5 UV/Visible Spectrophotometer (ALT) 48 h after transfection. The luciferase activity was calculated using the firefly-to-*Renilla* signal ratio and each LTR8B activity was normalized to the background negative control.

### Bioinformatic analyses

#### Bulk RNA-seq data analysis

The RNA-seq reads were aligned to the GRCh38/hg38 genome assembly and the GENCODE V39 transcriptome assembly separately using STAR v2.5.3a^56^ with the parameters --outFilterMultimapNmax 1 to only keep uniquely mapped reads in both alignments. Transcriptome alignments were quantified using RSEM v1.3.3 (ref. ^57^). Genomic alignments were combined to generate RPM signals for visualization. DEGs were defined by DESeq2 v1.38.2 (ref. ^58^) with default settings. For the quantification of individual retrotransposons, aligned reads were overlapped with retrotransposon annotations (RepeatMasker). Differentially expressed retrotransposons were defined by DESeq2 v1.38.2 using size factor estimated from gene count matrix.

#### Bulk ATAC-seq data analysis

Low-quality ATAC-seq reads (Phred score < 20) were removed and adaptors were trimmed using trim_galore v0.4.3 under paired-end mode. The reads were aligned to the GRCh38/hg38 genome assembly using Bowtie2 v2.3.3.1 (ref. ^59^) with the parameters -N 1 -L 25 -X 2000 --no-discordant --no-mixed. PCR duplicates were removed using Picard MarkDuplicates v2.9.0. To make the start site of each read represent the centre of the transposase binding^60^, the alignments were adjusted using MACS2 v2.1.0 (ref. ^61^) with the parameters --shift -75 --extsize 150.

S3norm^62^ was applied for signal normalization using the default setting (bin size, 20 bp). Peaks were called by MACS2 with normalized signal. Peaks called from all samples were merged and differential peaks were defined by DESeq2 v1.22.1 with the S3norm normalized read counts. Individual retrotransposons with differential chromatin accessibility were assigned by overlapping the retrotransposon coordinate with the differential peaks.

#### CUT&Tag data analysis

CUT&Tag reads were trimmed using trim-galore with the default parameters, followed by mapping to GRCh38/hg38 and the SARS-CoV-2 genome^21^ using bowtie2 v2.4.2 (--end-to-end --very-sensitive --no-mixed --no-discordant --no-overlap --no-dovetail --phred33 -I 10 -X 2000). SEACR v1.3 (ref. ^63^) was used to call H3K27ac peaks using ‘norm’ and ‘stringent’ modes for normalization and thresholding, respectively. SICER v1.0.2 was used to call H3K27me3 peaks. The corresponding IgG libraries were used as a control for peak calling. Peaks for the same histone modifications were merged for differential analysis with DEseq2 v1.28.0.

#### Analysis of snRNA-seq data

The snRNA-seq raw FASTQ files generated from the MGISEQ-2000RS platform were converted to 10x Cell Ranger-compatible format. The converted FASTQ files were mapped to GRCh38/hg38 pre-mRNA genome and the SARS-CoV-2 genome (SARS-CoV-2 isolate Wuhan-Hu-1, GenBank NC_045512.2)^21^ using Cell Ranger v6.0.1 with the default setting. The output was processed using Seurat v4.0.1 (ref. ^64^). Doublets (doublet score > 0.75) were removed using the computeDoubletDensity (*d* = 15) function from scDblFinder v.1.2.0 (ref. ^65^). We then applied standard filtering (nUMI ≥ 1,000, nGene ≥ 1,000, nUMI < 25,000, nGene < 4,000 and mitochondria ratio < 0.05) and removed clusters with no marker gene expression. The quality control statistics are listed in Supplementary Table 9. Nuclei that passed the filtering (*n* = 62,132) were normalized and transformed using the SCTransform function of Seurat with the glmGamPoi method. The nuclei were then subclustered and annotated based on manual curation of markers and reference to placenta marker databases^66–68^.

Genes that were differentially expressed between patients and controls were defined for each cluster using the FindMarker function of Seurat. CellPhoneDB v2.1.7 (ref. ^27^) was used to analyse receptor–ligand interactions using standard processing and subsampling of 8,000 cells was done for both the patient and control samples. Patient-specific receptor–ligand pairs were defined by *P* < 0.05, as calculated by CellPhoneDB, and significant upregulation of the ligand in patient cells from the snRNA-seq differential analysis.

For pseudo-bulk signal, raw reads of individual nuclei were split into separate files based on the cell barcode and aligned to the GRCh38/hg38 genome assembly using STAR v2.7.10b, as described in bulk RNA-seq data analysis. Aligned reads with the same UMI were removed using the UMI_Tools v1.1.2 dedup function^69^. Genomic alignments of all nuclei from the same cell type from patient or control samples were merged to generate the pseudo-bulk RPM signals for visualization. Individual retrotransposon analysis was performed as described in bulk RNA-seq data analysis.

#### Analysis of snATAC-seq data

The snATAC-seq raw FASTQ files generated from the MGISEQ-2000RS platform were converted to 10x Cell Ranger ATAC-compatible format. The converted FASTQ files were mapped to the GRCh38/hg38 assembly by Cell Ranger ATAC v2.1.0 with default settings. Mapped snATAC-seq was processed using ArchR v1.0.1 (ref. ^31^). Briefly, nuclei were filtered (nFrags ≥ 1,000, TSSEnrichment ≥ 4 and PromoterRatio ≥ 0.075) and clusters with low overall transcriptional-start-site signals were removed^24^. Nuclei that passed filter (*n* = 68,786) were clustered and annotated by label transfer from snRNA-seq and manual curation of marker genes. The quality control statistics are listed in Supplementary Table 9.

For peak analysis, pseudo-bulk signals were generated. Raw reads of individual nuclei were split into separate files based on the cell barcodes. Duplicated reads in each nucleus were removed using FastUniq v1.1 (ref. ^70^). Reads from each nucleus were aligned to the GRCh38/hg38 assembly using STAR v2.5.3a as described in bulk ATAC-seq data analysis. Genomic alignments of nuclei from the same cell type were merged to generate pseudo-bulk signals for peak calling with MACS2 v2.2.7.1 and visualization. Peaks called from each cell type were merged to generate a master peak set, which was used for Peak2GeneLinkage analysis with ArchR and differentially accessible peak analysis. To define cell type-specific differentially accessible peaks in patients versus controls, we conducted a one-sided Poisson test on the normalized pseudo-bulk reads count and significantly differentially accessible peaks were defined by fold change > 1.5, *P*adj < 0.01 and peak RPKM signal > 1 in the tested group. For visualization in a heatmap (Fig. 2d), differentially accessible peaks were filtered by pseudo-bulk RPKM > 10 in at least one cell type to retain most accessible peaks. Differentially accessible individual retrotransposons were defined by overlapping retrotransposons with differentially accessible peaks.

#### Retrotransposon subfamily analysis

We used our SAMA pipeline^36^ for retrotransposon subfamily quantifications in bulk and single-nucleus RNA-seq and ATAC-seq. Briefly, reads from each nucleus for single-nucleus data or from each sample for bulk data were mapped to the GRCh38/hg38 assembly using STAR v2.5.3a with the parameter --outFilterMultimapNmax 150. Reads with more than one best genomic alignment that is uniquely anchored to the same repeat subfamily were rescued. Rescued multi-aligned reads and uniquely aligned reads were collected for subfamily quantification and differential analysis.

#### Motif, GO and retrotransposons enrichment analysis

Motif analysis was conducted using HOMER^71^ (v4.9.1 for ATAC-seq peaks and v4.11 for CUT&Tag peaks) using whole genome as background. Gene ontology analysis was performed using Metascape v3.5 (ref. ^72^) and GREAT v4.0.4 (ref. ^73^). Subfamily enrichment of differentially expressed individual retrotransposons was calculated by the ratio of observed over expected counts of elements in each subfamily. The expected numbers were estimated by (*n* / *N*) × *X*, where *n* is the total number of elements of each subfamily, *N* is the total number of retrotransposons in the genome and *X* is the total number of differential retrotransposons. *P* values were calculated using a hypergeometric test.

#### ChIP–seq data analysis

For the TSC micro-ChIP–seq (single-end) dataset generated in this study, reads were aligned to the GRCh38/hg38 assembly using Bowtie v1.3.0 (ref. ^74^) with the parameters ‐v 3 ‐ m 1 ‐‐best ‐‐strata. For the public placenta ChIP–seq dataset (paired-end)^41,42^, the reads were aligned to the GRCh38/hg38 assembly using Bowtie2 v7.5.0 with the parameters ‐N 1 ‐L 25 ‐X 500 ‐‐no‐discordant ‐‐no‐mixed. Multi-aligned reads were removed. PCR duplicates were removed using Picard MarkDuplicates v2.23.4 for both datasets.

#### Hi-C data analysis

The TSC Hi-C reads were mapped to the GRCh38/hg38 assembly using Juicer v1.13 (ref. ^75^) with the parameter -s Arima. Significant interactions were called by Fit-Hi-C at 5-kb resolution with threshold *q* < 0.05. For virtual 4C analysis, reads that interacted with the bait (LTR8B elements of interest) and their flanking 2-kb regions were extracted and aligned to the genome with BWA v0.7.15 and the RPKM signal was calculated for visualization.

#### Sensitivity analysis

Sensitivity analysis was conducted on bulk RNA-seq and snRNA-seq to determine the impact of different clinical conditions on transcriptomic data. Patient samples were divided into the following clinical conditions: term (Cov2, Cov3, Cov4 and Cov7) versus pre-term (Cov1, Cov5 and Cov6), Caesarean section (Cov2, Cov3, Cov5, Cov6 and Cov7) versus natural delivery (Cov1 and Cov4) and hypertension (Cov7). For analyses of term versus pre-term and Caesarean section versus natural delivery, we split the samples based on the conditions and performed differential analysis using DESeq2 v1.22.1 (ref. ^58^) with default settings. For hypertension, we removed Cov7 from the snRNA-seq dataset and repeated differential gene and GO analysis using the same methods mentioned earlier.

#### Statistics and reproducibility

No statistical methods were used to pre-determine sample size. The investigators were not blinded to allocation during experiments and outcome assessment. The experiments were not randomized. All statistical analyses were done using a one-tailed Student’s *t*-test, two-tailed Student’s *t*-test, one-tailed Wilcoxon test, two-tailed Wald’s test, one-tailed hypergeometeric test, one-tailed MAST hurdle model or a one-tailed Poisson test. Details such as the exact *P* values, statistical tests and experimental replicates are indicated in the figures or figure legends.

### Reporting summary

Further information on research design is available in the Nature Portfolio Reporting Summary linked to this article.

## Online content

Any methods, additional references, Nature Portfolio reporting summaries, source data, extended data, supplementary information, acknowledgements, peer review information; details of author contributions and competing interests; and statements of data and code availability are available at 10.1038/s41556-023-01169-x.

## Supplementary information

Supplementary Information Supplementary Figs. 1 and 2 and legends.

Reporting Summary

Supplementary Tables 1–9 Supplementary Tables 1–9.

Supplementary Data 1 Source data for Supplementary Fig. 1c,d.

## Data Availability

All sequencing datasets generated in this study have been deposited at ArrayExpress under the accession ID E-MTAB-11749 and at the European Genome-phenome Archive under the accession ID EGAS00001006263. Published trophoblast epigenomic datasets were acquired from JGA under the accession IDs JGA000074 and JGA000117. Source data are provided with this paper. All other data supporting the findings of this study are available from the corresponding authors on reasonable request.

## Source data

Source Data Fig. 6 Statistical source data.
Source Data Fig. 7 Statistical source data.
Source Data Fig. 8 Statistical source data.
Source Data Extended Data Fig. 2 Statistical source data.
Source Data Extended Data Fig. 3 Statistical source data.
Source Data Extended Data Fig. 4 Statistical source data.
Source Data Extended Data Fig. 5 Statistical source data.
Source Data Extended Data Fig. 7 Statistical source data.
Source Data Extended Data Fig. 8 Statistical source data.
Source Data Extended Data Fig. 10 Statistical source data.

## Code availability

All code used in this study was previously published and no customized code was used in this manuscript.
