# Supplementary material for: Single-cell analysis reveals transcriptomic and epigenomic impacts on the maternal–fetal interface following SARS-CoV-2 infection
Source: Nat Cell Biol. 2023 Jul 3;25(7):1047–60. doi: 10.1038/s41556-023-01169-x (PMC10344786; doi:10.1038/s41556-023-01169-x)
Supplement: Supplementary file 1 — Supplementary Figs. 1 and 2 and legends. [file 41556_2023_1169_MOESM1_ESM.pdf]

nature cell biology

Article https://doi.org/10.1038/s41556-023-01169-x

Single-cell analysis reveals transcriptomic and epigenomic impacts on the maternal–fetal interface following SARS-CoV-2 infection

In the format provided by the authors and unedited

Supplementary Information

Single-cell analysis reveals transcriptomic and epigenomic impacts on the maternal-fetal interface upon SARS-CoV-2 infection

Lin Gao, Vrinda Mathur, Sabrina Ka Man Tam, Xuemeng Zhou, Ming Fung Cheung, Luyan Chan, Guadalupe Estrada-Gutiérrez, Bo Wah Leung, Sakita Moungmaithong, Chi Chiu Wang, Liona C. Poon, and Danny Leung

Supplementary Figure 1

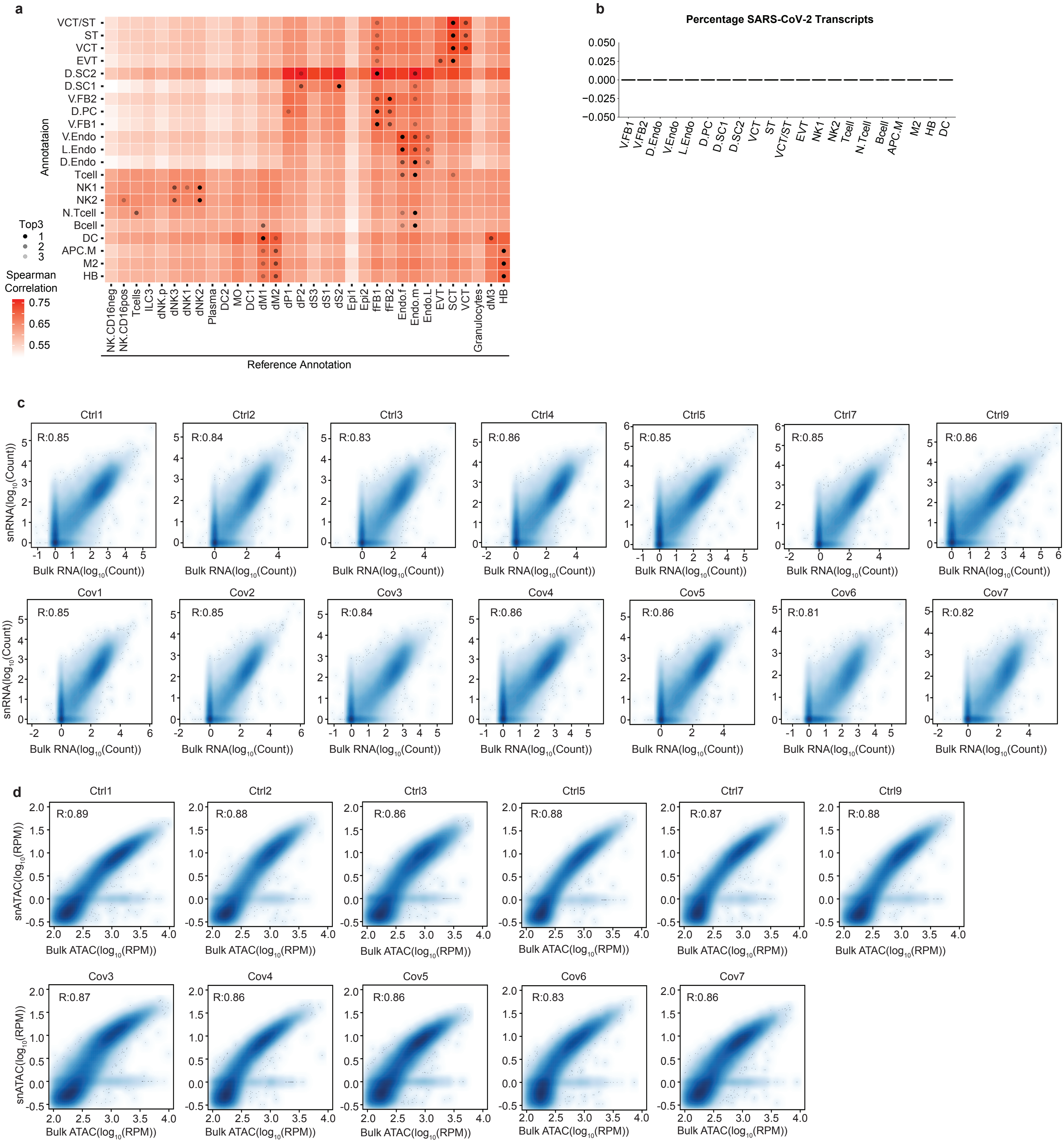

Supplementary Figure 1. Quality control for muti-omic datasets. a) Heatmap comparing our snRNA-seq cell type annotation to public annotation[18, 69]. The colour indicates the Spearman correlation coefficient and the black dots indicate the top three correlated annotations in each cell type. **b**) Violin plots showing the ratio of transcripts mapped to SARS-CoV-2 transcriptome in each cell type from the snRNA-seq. Note that all points reside near or on 0. **c**) Scatter plots showing the Spearman correlation between the gene counts in the pseudo-bulk snRNA-seq and the bulk RNA-seq. Genes were filtered for those have counts in either snRNA-seq or bulk RNA-seq. **d**) Scatter plots showing Spearman correlation between the signal in the pseudo-bulk snATAC-seq and the bulk ATAC-seq at all transcriptional start sites (TSS). TSSs were filtered for those with signal in either the snATAC-seq or the bulk ATAC-seq.

Supplementary Figure 2

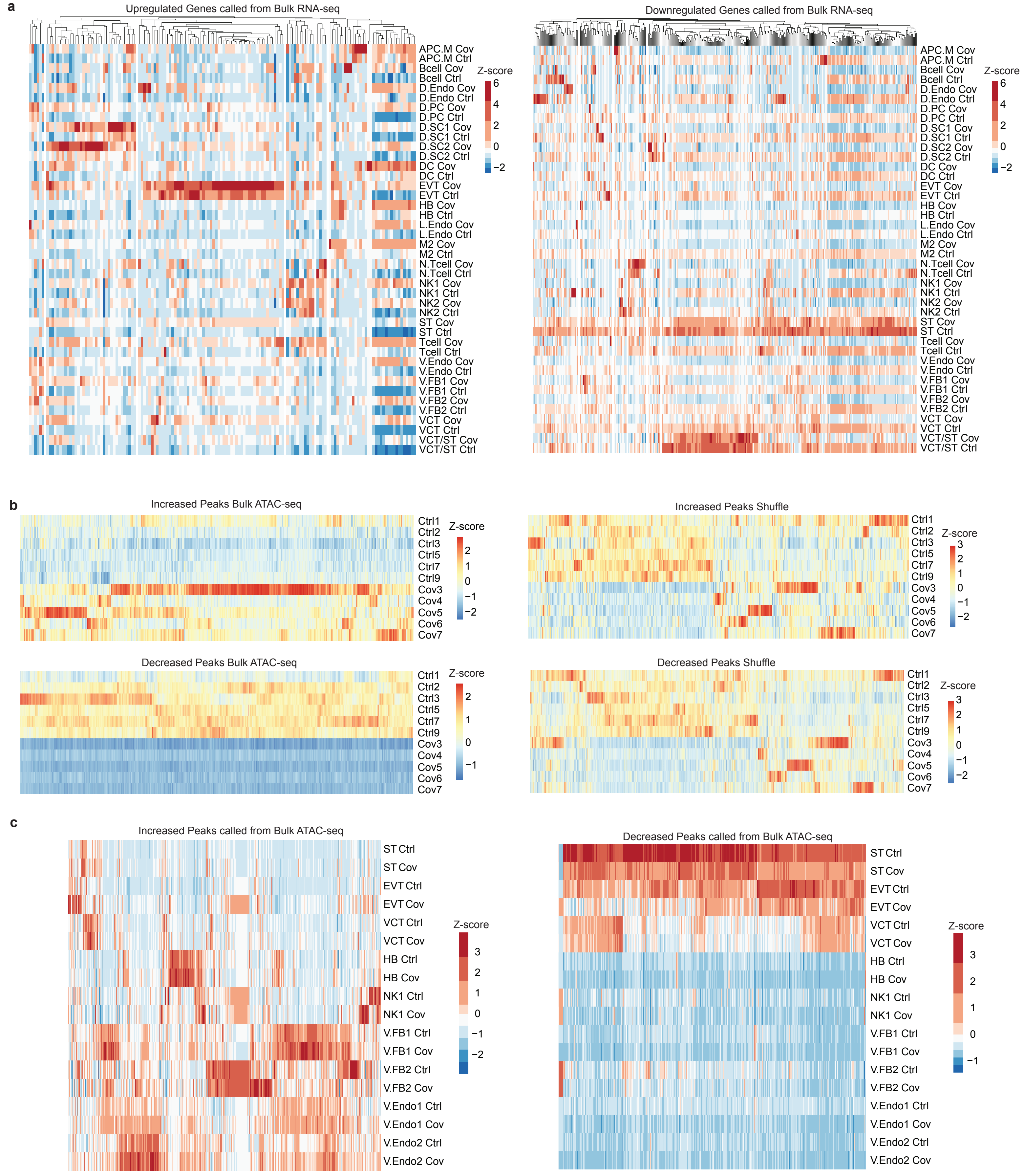

Supplementary Figure 2. Characterization between bulk and single-nuclei assays. a) Heatmaps showing expression of specific genes from each cell type from the snRNA-seq. Upregulated (left) (padj < 0.05 and log_2(fold change) > 1, n=211) and downregulated (right) (padj < 0.05 and log2(fold change) < -1, n=605) genes are defined from the bulk RNA-seq. Each row is a patient or control cell type from the snRNA-seq, each column is a differential gene called from the bulk RNA-seq and the colour represents the column z-score of expression calculated by Seurat. **b)** Heatmaps showing the bulk ATAC-seq signal of increased (top left) (padj < 0.01 and log_2(fold change) > 1, n=8,223) and decreased peaks (bottom left) (padj < 0.001 and log_2(fold change) < -1, n=7,142) in all samples compared to number-matched shuffle peaks control (right). The colour scale indicates Reads Per Kilobase per Million reads (RPKM) calculated column z-score. **c)** Heatmaps showing the chromatin accessibility at specific regions of each cell type from the snATAC-seq. Increased (left) (padj < 0.01 and log_2(fold change) > 1, n=8,223) and decreased (right) (padj < 0.001 and log_2(fold change) < -1, n=7,142) peaks are defined from the bulk ATAC-seq. Each row is a patient or control cell type from the snATAC-seq, each column is a differential peak called from the bulk ATAC-seq and the colour represents the column z-score of RPKM. Padj: adjusted two-tailed p-value from multiple testing using the Benjamini and Hochberg method calculated by DESeq2.
