# Supplementary material for: Single-cell analysis reveals transcriptomic and epigenomic impacts on the maternal–fetal interface following SARS-CoV-2 infection
Source: Nat Cell Biol. 2023 Jul 3;25(7):1047–60. doi: 10.1038/s41556-023-01169-x (PMC10344786; doi:10.1038/s41556-023-01169-x)
Supplement: Supplementary file 2 — Reporting Summary [file 41556_2023_1169_MOESM2_ESM.pdf]

nature portfolio

Corresponding author(s): Danny Leung & Liona C. Poon

Last updated by author(s): Apr 30, 2023

Reporting Summary

Nature Portfolio wishes to improve the reproducibility of the work that we publish. This form provides structure for consistency and transparency in reporting. For further information on Nature Portfolio policies, see our Editorial Policies and the Editorial Policy Checklist.

Statistics

For all statistical analyses, confirm that the following items are present in the figure legend, table legend, main text, or Methods section.

n/a	Confirmed	
☐	☒	The exact sample size (*n*) for each experimental group/condition, given as a discrete number and unit of measurement
☐	☒	A statement on whether measurements were taken from distinct samples or whether the same sample was measured repeatedly
☐	☒	The statistical test(s) used AND whether they are one- or two-sided<br>*Only common tests should be described solely by name; describe more complex techniques in the Methods section.*
☒	☐	A description of all covariates tested
☐	☒	A description of any assumptions or corrections, such as tests of normality and adjustment for multiple comparisons
☐	☒	A full description of the statistical parameters including central tendency (e.g. means) or other basic estimates (e.g. regression coefficient) AND variation (e.g. standard deviation) or associated estimates of uncertainty (e.g. confidence intervals)
☐	☒	For null hypothesis testing, the test statistic (e.g. *F*, *t*, *r*) with confidence intervals, effect sizes, degrees of freedom and *P* value noted<br>*Give P values as exact values whenever suitable.*
☒	☐	For Bayesian analysis, information on the choice of priors and Markov chain Monte Carlo settings
☒	☐	For hierarchical and complex designs, identification of the appropriate level for tests and full reporting of outcomes
☐	☒	Estimates of effect sizes (e.g. Cohen's *d*, Pearson's *r*), indicating how they were calculated

Our web collection on statistics for biologists contains articles on many of the points above.

Software and code

Policy information about availability of computer code

Data collection	No software was used to collect data.
Data analysis	STAR(v2.5.3a, v2.7.10b)<br>RSEM v1.3.3<br>DESeq2(v1.38.2, v1.22.1, v1.28.0)<br>Trim_galore v0.4.3<br>Bowtie2(v2.3.3.1, v2.4.2, v1.3.0, v7.5.0)<br>Bowtie v1.3.0<br>Picard MarkDuplicates (v2.9.0, v2.23.4)<br>MACS2 (v2.1.0, v2.2.7.1)<br>SEACR v1.3<br>FastUniq v1.1<br>ArchR v1.0.1<br>SICER v1.0.2<br>scDblFinder v1.2.0<br>MACS2 v2.2.7.1<br>10x Genomics Cellranger v6.0.1<br>10x Genomics Cellranger ATAC v2.1.0<br>Seurat v4.0.1<br>CellPhoneDB v2.1.7<br>UMI_Tools v1.1.2

ArchR v1.0.1
FastUniq v1.1
Subfamily Assignment Multiple Alignment (SAMA) pipeline
HOMER Motif Analysis (v4.9.1, v4.11)
Metascape v3.5
GREAT v4.0.4
Juicer v1.13
Fit Hi-C
BWA v0.7.15
Parameter details are described in the respective method sections.

For manuscripts utilizing custom algorithms or software that are central to the research but not yet described in published literature, software must be made available to editors and reviewers. We strongly encourage code deposition in a community repository (e.g. GitHub). See the Nature Portfolio guidelines for submitting code & software for further information.

Data

Policy information about availability of data

All manuscripts must include a data availability statement. This statement should provide the following information, where applicable:

- Accession codes, unique identifiers, or web links for publicly available datasets
- A description of any restrictions on data availability
- For clinical datasets or third party data, please ensure that the statement adheres to our policy

All sequencing datasets (snRNA-seq, snATAC-seq, CUT&Tag, bulk RNA-seq, bulk ATAC-seq, TSC Hi-C, TSC RNA-seq and TSC ChIP-seq) generated in this study have been deposited at ArrayExpress under the accession ID E-MTAB-11749 and at EGA under accession ID EGAS00001006263. Details of the generated datasets are in Supplementary Table 8. Genome assembly used is GRCh38/hg38 for all sequencing analysis. Additionally, the snRNA-seq and bulk RNA-seq were aligned to the SARS-CoV-2 genome (severe acute respiratory syndrome coronavirus 2 isolate Wuhan-Hu-1, GenBank NC_045512.2). Published trophoblast epigenomic datasets were acquired from JGA under accession IDs JGA000074 and JGA000117. Source data is provided with this study.

Human research participants

Policy information about studies involving human research participants and Sex and Gender in Research.

Reporting on sex and gender	14 pregnant females were recruited for this study, 7 COVID-19 positive patients and 7 healthy controls. Information regarding serial Ct values at and after diagnosis, demographic, clinical data and neonatal NPS/NPA RT-qPCR results were collected and are summarised in Extended Data Figure 1 and Supplementary table 1-3. All participants gave written informed consent to participate in the study. No compensation was provided to the participants for this study.
Population characteristics	Consecutive pregnant patients who tested positive for SARS-CoV-2 infection by RT-qPCR of a deep throat saliva (DTS) or nasopharyngeal swab (NPS) sample were enrolled during pregnancy or at time of delivery between 27 March 2020 and 24 January 2021. The median maternal age is 35 years and 37 years for control and patients respectively. All participants, both patients and controls, are of Asian descent. More clinical diagnosis and information are described in detail in Supplementary Table 1.
Recruitment	The patients were chosen using consecutive selection between 27 March 2020 and 24 January 2021. 7 patients with pregnancy beyond 24 weeks of gestation with positive RT-qPCR for SARS-CoV-2 during pregnancy were selected. 7 Uninfected women (as determined by negative RT-qPCR testing of DTS/NPS) who undergone an elective Cesarean section were recruited to donate the tissues to serve as uninfected controls for this study.
Ethics oversight	Approval for the study was obtained from the Joint Chinese University of Hong Kong - New Territories East Cluster Clinical Research Committee (CREC Ref. No. 2020.210)

Note that full information on the approval of the study protocol must also be provided in the manuscript.

Field-specific reporting

Please select the one below that is the best fit for your research. If you are not sure, read the appropriate sections before making your selection.

☒ Life sciences ☐ Behavioural & social sciences ☐ Ecological, evolutionary & environmental sciences

For a reference copy of the document with all sections, see nature.com/documents/nr-reporting-summary-flat.pdf

Life sciences study design

All studies must disclose on these points even when the disclosure is negative.

Sample size	No statistical methods were used to predetermine sample size. The sample size used in this study is similar to other studies with clinical samples. Our single-nucleus datasets have replicates with n=6 or above and our bulk assays have n=4 or above for each condition which is sufficient to calculate statistical significance. Samples size equals to the number of participant recruited in this study.

Data exclusions

No raw data uploaded to the data portal were excluded. For snRNA-seq and snATAC-seq analyses final count matrix, we excluded nuclei with poor quality and contamination (eg. low number of genes and high mitochondrial reads). Detailed filtering parameters were described in the relevant Methods section.

Replication

For snRNA-seq, maternal-fetal interface tissue from 7 healthy controls and 7 COVID-19 patients were performed. For snATAC-seq, maternal-fetal interface tissue from 7 healthy controls and 6 COVID-19 patients were performed. For bulk RNA-seq, maternal-fetal interface tissue from 7 healthy controls and 7 COVID-19 patients were performed. For bulk ATAC-seq, maternal-fetal interface tissue from 6 healthy controls and 5 COVID-19 patients were performed. For H3K27ac and H3K27me3 CUT&Tag, maternal-fetal interface tissue from 4 healthy controls and 5 COVID-19 patients were performed. Individual tissue samples collected from the 14 participants were used for the immunohistochemistry staining. All attempts at replication were successful, the results produced were highly reproducible. We generated epigenomic data for the trophoblast stem cell line (TSC) which are comparable to public epigenomic datasets used in this study.

Randomization

No randomization was performed in this study. This is a case-control study, conditions of samples were pre-established before sample collection. Covariates were assessed using sensitivity analyses to judge their influence on the statistical significance of our findings. Details are included in Extended data figures 2b, 2c, 6a and Supplementary Table 5. Experiments on TSC were performed on wildtype cells only, no randomization is needed.

Blinding

No blinding was performed in this study. This is a case control study, conditions of samples were pre-established before sample collection. Experiments on TSC were performed on wild type cell only, no blinding is needed.

Reporting for specific materials, systems and methods

We require information from authors about some types of materials, experimental systems and methods used in many studies. Here, indicate whether each material, system or method listed is relevant to your study. If you are not sure if a list item applies to your research, read the appropriate section before selecting a response.

Materials & experimental systems

n/a	Involved in the study
☐	☒ Antibodies
☐	☒ Eukaryotic cell lines
☒	☐ Palaeontology and archaeology
☒	☐ Animals and other organisms
☒	☐ Clinical data
☒	☐ Dual use research of concern

Methods

n/a	Involved in the study
☐	☒ ChIP-seq
☒	☐ Flow cytometry
☒	☐ MRI-based neuroimaging

Antibodies

Antibodies used

Immunohistochemical staining:
FLNB (ab282106, Abcam)(Dilution 1:5000) - https://www.abcam.com/filamin-b-antibody-epr24525-3-ab282106.html
PAPPA (ab174314, Abcam)(Dilution 1:200) - https://www.abcam.com/papp-a-antibody-9-ab52030.html
PSG9 (AP53483PU-N,Origene)(Dilution 1:100). - https://www.origene.com/catalog/antibodies/primary-antibodies/ap53483pu-n/psg9-center-rabbit-polyclonal-antibody
HRP (AP188P, Sigma-Aldrich)(Dilution 1:2000) - https://www.sigmaaldrich.com/HK/en/product/mm/ap188p

µChIP-seq Antibodies:
H3K27ac (AM39133, Active Motif)(Dilution 1:90) - https://www.activemotif.com/catalog/details/39133/histone-h3-acetyl-lys27-antibody-pab
H3K4me3 (AM39915, Active Motif)(Dilution 1:90)- https://www.activemotif.com/catalog/details/39915/histone-h3-trimethyl-lys4-antibody-pab-1
H3K4me1 (AM91289, Active Motif)(Dilution 1:90)- https://www.activemotif.com/catalog/details/91289/abflex-histone-h3k4me1-antibody-rab.

CUT&Tag Antibodies:
H3K27me3 (AM39155, Active Motif)(Dilution 1:50) - https://www.activemotif.com/catalog/details/39155
H3K27ac (AM39133, Active Motif)(Dilution 1:50) - https://www.activemotif.com/catalog/details/39133/histone-h3-acetyl-lys27-antibody-pab
Rabbit IgG antibody (I5006, Sigma-Aldrich)(Dilution 1:50) - https://www.sigmaaldrich.com/HK/en/product/sigma/i5006
Donkey anti-rabbit IgG secondary antibody (ab6701, Abcam)(Dilution 1:100) - https://www.abcam.com/products/secondary-antibodies/donkey-rabbit-igg-hl-ab6701.html

Validation

FLNB - Validated by manufacturer using WB, ICC/IF, IHC and Flow Cytometry (https://www.abcam.com/filamin-b-antibody-epr24525-3-ab282106.html)
PAPPA - Validated by manufacturer using IHC-P and IHC-Fr (https://www.abcam.com/papp-a-antibody-9-ab52030.html)
PSG9 - Validated by manufacturer using IHC and WB (https://www.origene.com/catalog/antibodies/primary-antibodies/ap53483pu-n/psg9-center-rabbit-polyclonal-antibody)
HRP - Validated by manufacturer using ELISA and WB (https://www.sigmaaldrich.com/HK/en/product/mm/ap188p)
H3K27ac - validated by ChIP, ChIP-seq, ICC/IF, WB, and CUT&Tag (https://www.activemotif.com/catalog/details/39133/histone-h3-acetyl-lys27-antibody-pab)

H3K4me3 - validated by ChIP, ChIP-seq, ICC/IF and WB (https://www.activemotif.com/catalog/details/39915/histone-h3-trimethyl-lys4-antibody-pab-1)
H3K4me1 - validated by WB (https://www.activemotif.com/catalog/details/91289/abflex-histone-h3k4me1-antibody-rab.)
H3K27me3 - validated by ChIP, ChIP-seq, ICC/IF, WB, and CUT&Tag (https://www.activemotif.com/catalog/details/39155)
Donkey anti-rabbit IgG secondary antibody - validated by WB, ELISA, IP, Conjugation, IHC-P, IHC-Fr, Immunodiffusion and ICC/IF (https://www.abcam.com/products/secondary-antibodies/donkey-rabbit-igg-hl-ab6701.html)

Detailed product information can be found on their respective websites.

Eukaryotic cell lines

Policy information about cell lines and Sex and Gender in Research

Cell line source(s)	The human trophoblast stem cell line was a gift from Dr Pengtao Liu, School of Biomedical Sciences, the University of Hong Kong. It was derived by, published by, and obtained directly from Dr Liu (Gao X. et.al, Nature Cell Biol., 2019, doi: 10.1038/s41556-019-0333-2, PMID: 31160711). His group is the original source of this cell line.
Authentication	No additional authentication was done
Mycoplasma contamination	Mycoplasma tested negative
Commonly misidentified lines (See ICLAC register)	No commonly misidentified cell lines were used

ChIP-seq

Data deposition

☒ Confirm that both raw and final processed data have been deposited in a public database such as GEO.

☒ Confirm that you have deposited or provided access to graph files (e.g. BED files) for the called peaks.

Data access links *May remain private before publication.*	https://apc01.safelinks.protection.outlook.com/?url=https%3A%2F%2Fwww.ebi.ac.uk%2Farrayexpress%2Fexperiments%2FE-MTAB-11749&data=05%7C01%7Ckmtam%40connect.ust.hk%7C408b755c13ac48875f4b08da4eabbfe6%7C6c1d415239d044ca88d9b8d6ddca0708%7C1%7C0%7C637908796836635809%7CUnknown%7CTWFpbGZsb3d8eyJWIjoiMC4wLjAwMDAiLCJQIjoiV2luMzIiLCJBTiI6Ik1haWwiLCJXVCI6Mn0%3D%7C3000%7C%7C%7C&sdata=FCVzNUChL4NT9CfPQAxGp2iwSnPYjZtIU%2FCaD7YU1zI%3D&reserved=0<br><br>wwwdev.ebi.ac.uk/ega/studies/EGAS00001006263
Files in database submission	TSC_H3K27ac_uChIP.fastq.gz<br>TSC_H3K4me1_uChIP.fastq.gz<br>TSC_H3K4me3_uChIP.fastq.gz<br>TSC_input_uChIP.fastq.gz<br>TSC_H3K27ac_uChIP.bw<br>TSC_H3K4me1_uChIP.bw<br>TSC_H3K4me3_uChIP.bw<br>TSC_input_uChIP.bw
Genome browser session (e.g. UCSC)	http://hgw1.soe.ucsc.edu/s/SabrinaTam/SARS-CoV-2_MFI

Methodology

Replicates	Trophoblast stem cell ChIP-seq dataset was generated with single replicates
Sequencing depth	ChIP-seq libraries were sequenced to have greater than 20 million uniquely mapped reads
Antibodies	H3K27ac (AM39133, Active Motif), H3K4me3 (AM39915, Active Motif), and H3K4me1 (AM91289, Active Motif).
Peak calling parameters	macs2 callpeak –nomodel —keep-dup all -q 0.05.
Data quality	ChIP-seq signal was compared to previous published datasets for the same cell type.
Software	Bowtie v1.3.0<br>Picard MarkDuplicates v2.23.4<br>MACS2 v2.2.7.1
